# Epidemiology of *E. coli* in Cystic Fibrosis Airways Demonstrates the Capacity for Persistent Infection but Not Patient-Patient Transmission

**DOI:** 10.3389/fmicb.2020.00475

**Published:** 2020-03-20

**Authors:** Conrad Izydorczyk, Barbara Waddell, Brett D. Edwards, Jasper Greysson-Wong, Michael G. Surette, Ranjani Somayaji, Harvey R. Rabin, John M. Conly, Deirdre L. Church, Michael D. Parkins

**Affiliations:** ^1^Department of Microbiology, Immunology and Infectious Diseases, Cumming School of Medicine, University of Calgary, Calgary, AB, Canada; ^2^Department of Medicine, Cumming School of Medicine, University of Calgary, Calgary, AB, Canada; ^3^Department of Biochemistry and Biomedical Sciences, McMaster University, Hamilton, ON, Canada; ^4^Snyder Institute for Chronic Diseases, Cumming School of Medicine, University of Calgary, Calgary, AB, Canada; ^5^Department of Pathology and Laboratory Medicine, Cumming School of Medicine, University of Calgary, Calgary, AB, Canada; ^6^Alberta Health Services, Calgary, AB, Canada

**Keywords:** cystic fibrosis, *Escherichia coli*, transmission, epidemiology, genomics, whole genome sequencing, natural history, infection

## Abstract

*Escherichia coli* is frequently isolated from the respiratory secretions of cystic fibrosis (CF) patients yet is not considered a classical CF pathogen. Accordingly, little is known about the natural history of this organism in the CF airways, as well as the potential for patient-to-patient transmission. Patients attending the Calgary Adult CF Clinic (CACFC) between January 1983 and December 2016 with at least one *E. coli*-positive sputum culture were identified by retrospective review. Annual *E. coli* isolates from the CACFC biobank from each patient were typed by pulsed-field gel electrophoresis (PFGE) and isolates belonging to shared pulsotypes were sequenced. Single nucleotide polymorphism (SNP) and phylogenetic analysis were used to investigate the natural history of *E. coli* infection and identify potential transmission events. Forty-five patients with *E. coli*-positive sputum cultures were identified. Most patients had a single infection episode with a single pulsotype, while replacement of an initial pulsotype with a second was observed in three patients. Twenty-four had *E. coli* recovered from their sputum more than once and 18 patients had persistent infections (*E. coli* carriage >6 months with ≥3 positive cultures). Shared pulsotypes corresponded to known extraintestinal pathogenic *E. coli* strains: ST-131, ST-73, and ST-1193. Phylogenetic relationships and SNP distances among isolates within shared pulsotypes were consistent with independent acquisition of *E. coli* by individual patients. Most recent common ancestor date estimates of isolates between patients were inconsistent with patient-to-patient transmission. *E. coli* infection in CF is a dynamic process that appears to be characterized by independent acquisition within our patient population and carriage of unique sets of strains over time by individual patients.

## Introduction

Progressive airways disease due to persistent and recurrent bacterial infection is the primary cause of morbidity and mortality in patients with cystic fibrosis (CF) (Ratjen et al., 2015). Accordingly, many studies have investigated infection dynamics and identified associations between infections with several specific organisms and a poor prognosis (Zemanick and Hoffman, 2016). Not surprisingly, the focus of many such studies has been on “classical” CF pathogens – prevalent organisms such as *Pseudomonas aeruginosa*, *Staphylococcus aureus*, and the *Burkholderia cepacia* complex. However, increasingly we are cognizant that the breadth of organisms capable of infecting CF airways is more diverse. As such, an increasing proportion of CF microbiological studies has shifted to include “non-classical” organisms such as *Streptococcus* sp., *Prevotella* sp., and *Escherichia coli*.

*Escherichia coli* is a Gram-negative bacterium that includes both commensal and pathogenic strains. It is responsible for a high burden of human disease, including gastrointestinal disease, genito-urinary infections, sepsis, and meningitis, and can be broadly divided into pathotypes based on the type of disease manifested (Donnenberg, 2015). Strains that cause disease at sites other than the gastrointestinal tract are broadly termed extraintestinal pathogenic *E. coli* (ExPEC) and include those that cause genito-urinary tract infections.

While not typically considered a respiratory pathogen, *E. coli* can cause respiratory illness, including ventilator-associated pneumonia (VAP). Indeed, recent studies have observed that *E. coli* and other Enterobacteriaceae may have overtaken *P. aeruginosa* as the predominant cause of VAP (Peleg and Hooper, 2010; Fihman et al., 2015). Furthermore, community-acquired pneumonia caused by *E. coli*, along with other Gram-negative bacilli, is associated with an elevated risk of severe disease and mortality (Marrie et al., 1998; Arancibia et al., 2002; Falguera et al., 2009; Ruiz et al., 2010). Despite these trends, little is known about the role of *E. coli* in CF lung infections. Only a single study has investigated the microbiological characteristics and epidemiology of *E. coli* in CF, in which the authors observed a background prevalence of *E. coli* in CF of approximately 25% (Barillova et al., 2014). Here they further observed that CF-associated strains typically belonged to the B2 phylogroup, which itself mainly consists of ExPEC strains.

The potential for patient-to-patient infection transmission in CF was first recognized in the 1980s with the identification of transmissible “epidemic” strains of *B. cenocepacia* (LiPuma et al., 1990; Smith et al., 1993). Since then, numerous studies have identified or hypothesized instances of patient-to-patient transmission in CF of various pathogens, including *Burkholderia* sp. (Lieberman et al., 2011), *P. aeruginosa* (Denton et al., 2002; Marvig et al., 2015), and *Mycobacteroides* (formerly *Mycobacterium*) *abscessus* complex (Bryant et al., 2013). Many of these comprise epidemic strains, most notably *P. aeruginosa*, which are shared among many CF patients and often represent CF-specific lineages (Parkins et al., 2018). However, studies to date have neither investigated the potential of *E. coli*, as a non-classical CF pathogen, for patient-to-patient transmission, nor for the existence of epidemic lineages – an important consideration given its evolved role as a human pathogen.

In this work, we investigated the genetic relationships among *E. coli* isolates between CF patients attending the Calgary Adult CF Clinic in Calgary, Canada to understand the natural history of *E. coli* airways infection in CF and to determine if patient-to-patient transmission may have occurred.

## Results

### Natural History of *E. coli* Infection in CF

We identified 45/366 patients (∼12.3%) from our clinic who had experienced *E. coli* infections between January 1983 and December 2016. Within the CACFC biobank, there were 310 individual *E. coli* isolates. Of the cohort, 21/45 patients (∼47%) had a single *E. coli*-positive sputum culture, while 24 patients (∼53%) had *E. coli* recovered from their sputum more than once. Eighteen patients met our criteria for persistent infection (defined as having ≥3 *E. coli* positive sputum cultures with carriage over 6 months). To understand the natural history of infection, we sought to determine whether serially collected isolates from patients with multiple *E. coli*-positive sputum cultures were related and represented colonization by a single or multiple clonal lineage(s) over time.

Focusing on incident and last infection isolates, along with intermittent isolates collected 1–3 years apart in patients with multiple *E. coli*-positive sputum cultures, we typed 89 isolates (18 transient, 71 persistent) from 31/45 patients (∼69%) (median 2 isolates/patient, range 1–10), including all 18 with persistent infections, by PFGE (Supplementary Figure S1). Of these 31 patients, 23 had at least two *E. coli*-positive sputum cultures. Isolates from the remaining 14/45 patients (all with transient infections) were either not found (12 isolates from 12 patients), could not be recovered from frozen cultures (1 isolates from 1 patient), or could not be typed by PFGE (1 isolates from 1 patient). There were no significant differences in patient age, pancreatic status, *P. aeruginosa* isolation at first time of *E. coli* isolation, or F508 homozygous genotype status among included and excluded patients (data not shown). The clinical impact of *E. coli* infection within these patients is reported elsewhere (Edwards et al., 2019).

**FIGURE 1:**
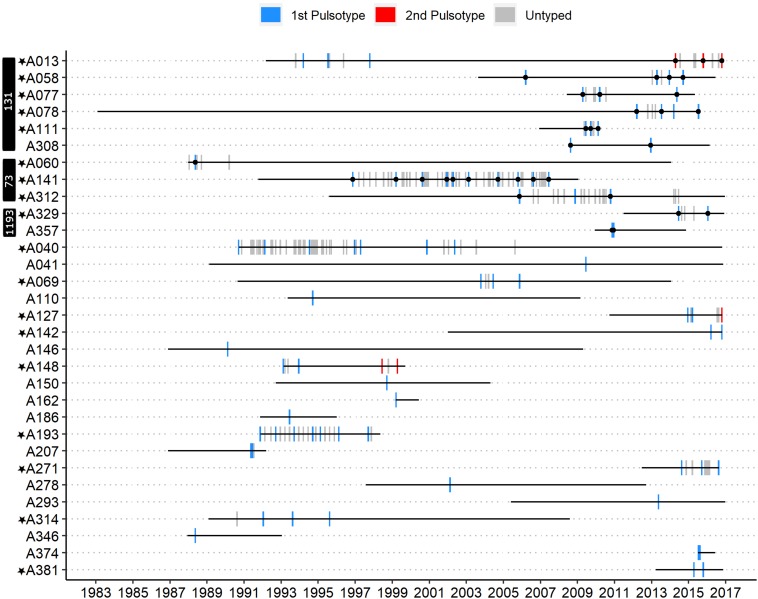
Timeline of *E. coli*-positive sputum cultures for all patients who had at least one isolate typed by PFGE. Blue bars represent the first pulsotype recovered per patient, and red bars the second pulsotype. Gray bars represent *E. coli*-positive sputum cultures that were not typed by PFGE. Each bar corresponds to the month in which each culture was collected. Black circles overlapping vertical bars represent sequenced isolates. Patients marked with a black star met the criteria for persistent infection. MLST sequence type of sequenced isolates is indicated in white text on the black vertical bars.

A single PFGE pulsotype was recovered from all but 3/31 patients (Figure 1). Patients A013 and A148 each had two distinct persistent infection episodes. A patient was inferred to have multiple distinct episodes if the time between their last *E. coli* positive sputum culture from a first episode to the first *E. coli* positive sputum culture of a subsequent episode spanned multiple years (>1), contained multiple *E. coli* negative sputum cultures, and a unique pulsotype was recovered in the subsequent episode. In fact, 32 *E. coli-*negative cultures collected over ∼16.6 years separated patient A013’s two persistent infection episodes and 28 *E. coli*-negative cultures collected over ∼4.5 years separated patient A148’s two persistent infection episodes (Figure 1). In both of these patients, a second pulsotype unrelated to the first (<80% identical banding pattern, >3 band differences) was recovered in the second episode. Patient A127, in contrast, had only seven *E. coli*-negative sputum cultures separating their first and second episodes approximately 1.6 years apart, in which a second unrelated pulsotype was identified. In addition, patient A312 had a suspected second infection episode approximately 3.4 years after their first (with 14 *E. coli-*negative sputum cultures in between), but we were unable to type isolates from the second episode with PFGE and so are unsure of its relation to the first. While it is possible that a second recovered pulsotype could result from hypermutation of the original pulsotype, we did not observe elevated mutation rates relative to other isolates in our collection in sequenced isolates from patients A013 and A312. Similarly, we did not observe any frameshift/non-sense mutations in any genes known to be associated with *E. coli* hypermutation in these two patients (Supplementary Tables 5, 6) (Oliver and Mena, 2010). Patient A312’s two sequenced isolates had a single missense mutation in *uvrD* and two missense mutations in *mutY* each, but these were common across all ST-73 isolates in our collection. As we did not sequence any isolates from patients A127 or A148, we cannot be certain that the second pulsotype recovered from these patients were not due to hypermutation of their original pulsotypes, although this was deemed exceedingly unlikely. Patient A058 had two persistent infection episodes approximately 6.8 years apart, each with isolates belonging to pulsotype cluster C, but with 30 *E. coli*-negative sputum cultures in between and ≤3 differing bands, these were attributed to independent acquisitions of a common clone (ST-131, see below). At no time did we recover the original pulsotype after detecting a second pulsotype in a patient. However, since we did not type all isolates from all patients, it is possible that a) some patients had a second/subsequent pulsotype that was not detected, and b) that we could have detected the original pulsotype with denser typing.

**FIGURE 2:**
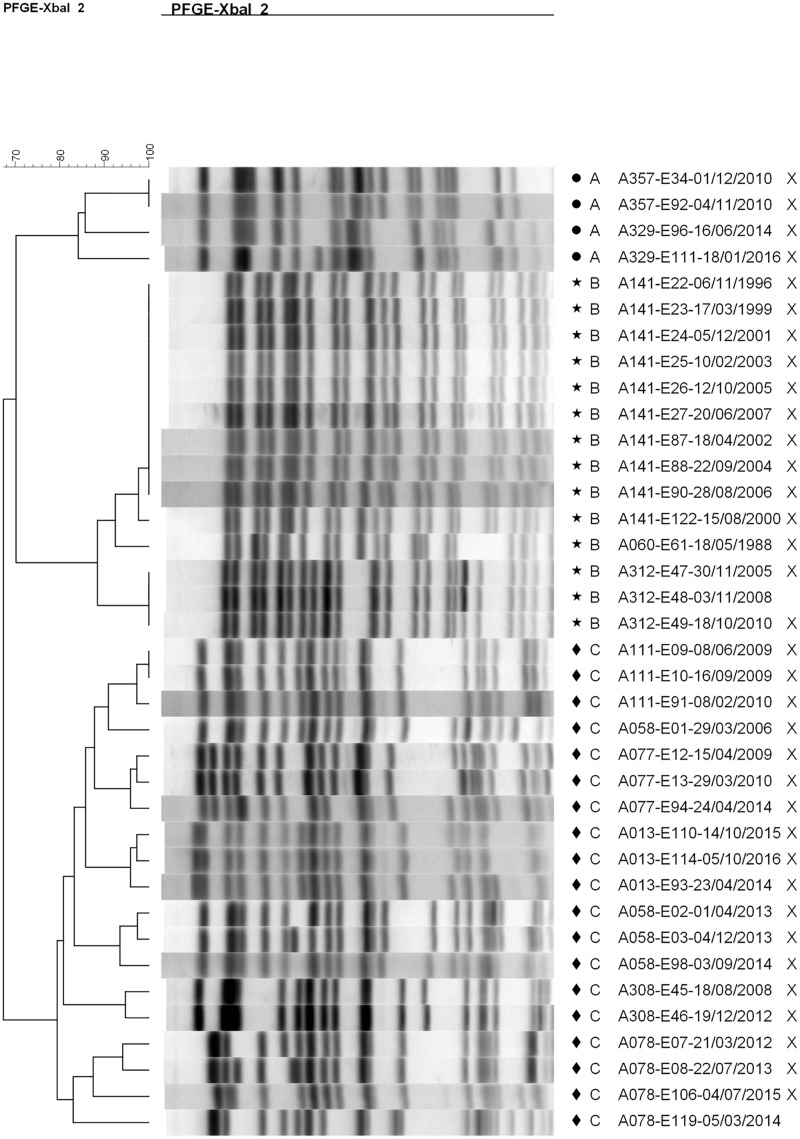
Dendrogram showing pulsotype relationships between isolates belonging to shared pulsotypes. A (circle) = pulsotype cluster A (ST-1193), B (star) = pulsotype cluster B (ST-73), and C (diamond) = pulsotype cluster C (ST-131). Isolates sequenced in this study are marked with an “X”. The dendrogram was generated using the UPGMA method with 2% tolerance. Isolate names are coded as “patient identifier”-“isolate number”-“culture date of isolate (dd-mm-yyyy)”.

Most patients (20/31, 65%) and most episodes of *E. coli* infection within our cohort were associated with isolates belonging to unique pulsotypes (52/89 typed isolates). The remaining 11 patients (35%) were infected with isolates belonging to one of three shared pulsotypes (Figure 2). Collectively over these 3 pulsotypes, patients were represented by a median of 3 isolates (range 1–10) collected over a mean time period of 4.15 years (range 0.07–10.62); Table 1 presents details on each pulsotype. One patient (A013) with two persistent infection episodes had pulsotype C recovered only from their second episode, and a second patient (A058) had two distinct persistent infection episodes both with isolates belonging to pulsotype C (Figure 1).

**TABLE 1:** Summary of shared pulsotypes clusters.

Pulsotype cluster	MLST sequence type	Number of isolates in cluster	Number of patients in cluster	Median number of isolates/patient (range)	Approximate timespan of isolates* (years)
A	1193	4	2	2 (2)	5.2
B	73	14	3	3 (1–10)	22.5
C	131	19	6	3 (2–4)	10.5

* Approximate timespan of isolates refers to the approximate number of years over which isolates belonging to the indicated pulsotype/MLST sequence type were collected, across all patients.

Whole-genome sequencing (Supplementary Figure S2) and *in silico* multi-locus sequence typing of 35 isolates (Figure 1) identified the three shared pulsotypes as corresponding to three globally prevalent, ExPEC sequence types: ST-1193 (A), ST-73 (B), and ST-131 (C). *In silico* PCR typing of the 18 ST-131 isolates further identified all as belonging to the C1 subgroup. In particular, ST-131 is highly prevalent in the Calgary region and associated with carriage of extended-spectrum beta-lactamases (ESBLs) (Pitout et al., 2009; Peirano et al., 2010, 2012; Peirano and Pitout, 2014); three patients (A077, A162, and A374) had infections with ESBL-producing *E. coli* but only patient A077 had any isolates sequenced. The clinical impact of infection of these patients by ESBL-producing *E. coli* has previously been reported (Edwards et al., 2019).

**FIGURE 3:**
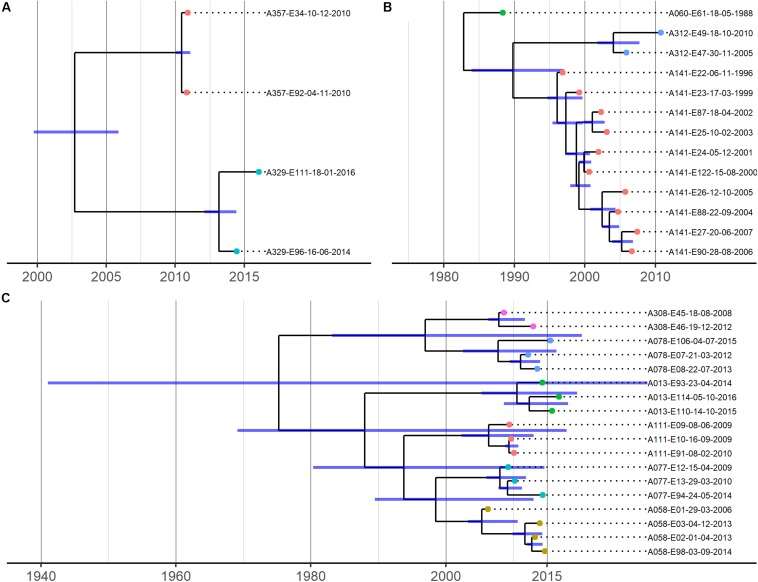
Time-calibrated phylogenies for 3 shared pulsotype clusters/STs. **(A)** = pulsotype cluster A (ST-1193), **(B)** = pulsotype cluster B (ST-73), **(C)** = pulsotype cluster C (ST-131). Blue bars represent 95% HPD intervals. Phylogenies were estimated using BEAST with a strict clock model with the substitution rate set to 4.03 × 10^–7^ **(A)** or uncorrelated relaxed clock models **(B,C)**. Isolate names are coded as “patient identifier”-“isolate number”-“culture date of isolate (dd-mm-yyyy)”. Isolates belonging to the same patient are further represented by same-colored dots at the tips of the corresponding branches.

The phylogenetic relationships between isolates from most patients were consistent with infection by a single strain (Figure 3). However, clear instances of within-patient *E. coli* diversity were also evident from the non-chronological ordering of sequentially collected isolates from individual patients and intra-patient SNP distances (Supplementary Tables 3–5), which were too large to represent sequential evolution of a single lineage over time given our substitution rate estimates (see below). For example, patient A141’s sequential isolates were not chronologically related in Figure 3B, with their 2002/2003 isolates sharing a common ancestor with a branch leading to their 2000/2001 and later isolates. Similarly, 17 core SNPs were observed to separate patient A013’s 2015 and 2016 isolates, and 46 core SNPs separated patient A312’s 2010 and 2015 isolates. As we were limited to isolates found in the CACFC biobank, and only 1 isolate was collected per time point per patient during clinic visits, we were unable to determine whether this observed diversity represents simultaneous infection by multiple strains or strain replacement over time.

### *E. coli* Colonization Is Characterized by Patient Carriage of Unique Sets of Strains

To understand whether the sharing of STs between patients could represent patient-to-patient transmission or independent infections of locally prevalent but only distantly related strains, we investigated the phylogenetic relationships and SNP distributions between isolates within each ST. For each ST, we assessed phylogenetic relatedness by constructing core SNP phylogenies and quantified genetic relatedness by comparing the number of SNPs identified among and between isolates from different patients. We further estimated divergence dates of isolates between patients using Bayesian phylogenetic reconstruction and compared these dates with epidemiological data to infer whether a transmission event may have taken place.

Recombination-corrected SNP phylogenies were constructed separately for each ST (Figure 3). These phylogenies revealed that isolates from individual patients were more closely related to each other than to isolates from other patients and formed patient-specific clades. Isolates from individual patients clustered into short-branching clades, with long branches separating isolates from different patients. These deep phylogenetic divisions are consistent with the independent acquisition of strains by each patient (Figure 1). In all cases, the genetic diversity among isolates of individual patients was independently derived based on our data analysis; patient-specific clades were derived from long branches ancestral to clades found in other patients. Mashtree phylogenies revealed that our CF genomes did not cluster together within the 3 STs but were scattered throughout, indicating a lack of evidence for CF-specific lineages (Supplementary Figures S3–S5).

**FIGURE 4:**
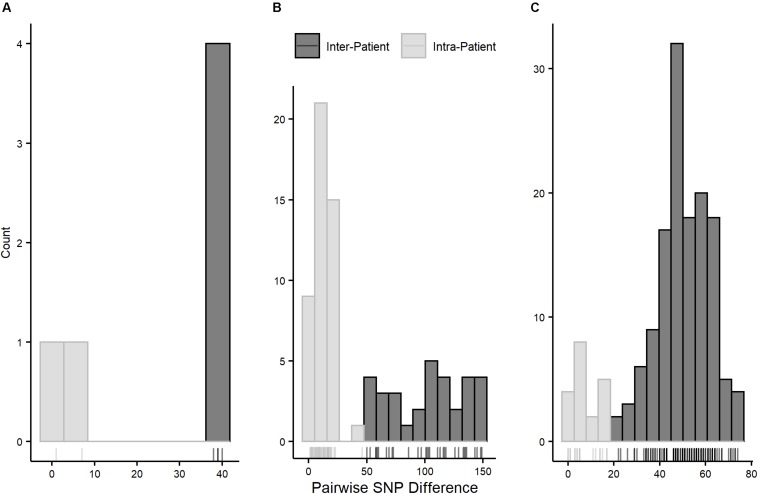
Pairwise SNP distance distributions by pulsotype cluster/ST. **(A)** = pulsotype cluster A (ST-1193); bin size = 5 SNPs, **(B)** = pulsotype cluster B (ST-73); bin size = 10 SNPs, **(C)** = pulsotype cluster C (ST-131); bin size = 5 SNPs. Intra-patient distances are in gray and inter-patient distances are in black.

Pairwise SNP distances for all three STs followed multinomial distributions with smaller distances within than between patients (Figure 4). Pairwise intra-patient SNP distances were always smaller than distances between patients regardless of the time between collection dates of compared isolates, and no overlap between intra- and inter-patient distances was observed within STs (Supplementary Tables 3–5). Overlap of intra- and inter-patient SNP distances between STs was observed due to 46 SNPs separating patient A312’s isolates (both ST-73) collected approximately 5 years apart. However, this distance is too great to represent direct evolution of the earlier lineage to the later given current substitution rate estimates in *E. coli* (see below), suggesting this SNP difference is due to carriage of independently evolving sub-lineages.

Divergence dates estimates of the most recent common ancestors (MRCAs) of isolates from different patients are shown in Figure 3 and Supplementary Table 6. Mean substitution rates for STs 131 and 73 were estimated to be 2.63 × 10^–7^ SNPs/site/year (95% HPD 7.65 × 10^–8^ to 4.81 × 10^–7^) and 8.55 × 10^–7^ SNPs/site/year (95% HPD 3.57 × 10^–7^ to 1.44 × 10^–6^), respectively, and were consistent with previous estimates for these STs (Reeves et al., 2011; Stoesser et al., 2016). Due to the small sample size, we were unable to estimate a substitution rate for ST-1193 and used a previously published rate of 4.03 × 10^–7^ SNPs/site/year for divergence date estimation (Johnson et al., 2019). In all pairwise patient comparisons, divergence date estimates of the MRCA of the patients’ isolates significantly predated the dates of first colonization of either patient, suggesting patient-to-patient transmission was exceedingly unlikely.

### Limited Signal for Pathoadaptation of *E. coli* to the CF Lung Environment

The identification of genes with multiple independent mutations (i.e. multi-mutated loci), as well as a higher ratio of non-synonymous to synonymous mutations relative to genes with only single mutations, has recently been used to identify candidate pathoadaptive genes in *P. aeruginosa* (Caballero et al., 2015) and *Burkholderia multivorans* (Diaz Caballero et al., 2018) infecting individuals with CF. Thus, we applied a similar approach to identify genes potentially involved in the adaptation of *E. coli* to the CF lung environment.

We identified 226, 285, and 45 total segregating SNPs among our ST-131, ST-73, and ST-1193 isolates (Supplementary Tables 8–10). Among these, we identified 12 and 21 loci containing ≥2 SNPs for ST-131 and ST-73, respectively (Table 2), suggesting that these loci may be involved in the adaptation of *E. coli* to the CF lung environment. No multi-mutated loci were identified among the segregating SNPs for ST-1193. However, a comparison of the frequencies of non-synonymous and synonymous mutations among multi-mutated loci and loci with only a single mutation did not reveal elevated rates of non-synonymous mutations among multi-mutated loci for neither ST-131 (chi-square test, *P* > 0.05) nor ST-73 (chi-square test, *P* > 0.05). Multi-mutated loci included a variety of proteins, including a number of hypothetical proteins and intergenic regions. Notably, two iron-acquisition loci (DR76_RS08305, DR76_RS14760) and a type-VI secretion system component (locus DR76_RS17725) all had multiple mutations among ST-73 isolates. Locus DR76_RS08305, which encodes the ferric aerobactin receptor IutA and has previously been found carried on a plasmid (Nash et al., 2010), had 5 unique SNPs, including two alternative bases relative to the reference at one site (three bases total). Aerobactin is a siderophore and important virulence factor in *E. coli* (Gao et al., 2012). Similarly, the DR76_RS14760 locus, which encodes the yersiniabactin polyketide synthase HMWP1 involved in production of the yersiniabactin siderophore (Miller et al., 2002), had two SNPs. Iron acquisition plays an important role in the virulence of uropathogenic ExPEC strains (Gao et al., 2012).

**TABLE 2:** Loci with ≥2 mutations (multi-mutated loci).

ST	Locus	Number of SNPs	Gene product
131	EC958_RS00830	2	ATP-dependent helicase HrpB
131	EC958_RS01890	2	Bifunctional 3-(3-hydroxy-phenyl)propioinate/3-hydroxycinnamic acid hydroxylase
131	EC958_RS04915	2	Tail fiber protein
131	EC958_RS07360–	2	Intergenic region
	EC958_RS07365		
131	EC958_RS11115	4	Ail/Lom family outer membrane beta-barrel protein
131	EC958_RS11380	2	tRNA-Ser
131	EC958_RS12105	2	Tyrosine-protein kinase Wzc
131	EC958_RS16810	4	IS66 family transposase
131	EC958_RS16955–	2	Intergenic region
	EC958_RS16960		
131	EC958_RS21030	2	Autotransporter adhesin Ag43
131	EC958_RS22500	2	6-phosphofructokinase
131	EC958_RS23010	2	5S ribosomal RNA
73	DR76_RS01390	2	Autotransporter outer membrane beta-barrel domain-containing protein
73	DR76_RS02050	2	Hypothetical protein
73	DR76_RS03475	2	Arylsulfatase
73	DR76_RS08000	2	Capsular biosynthesis protein
73	DR76_RS08305	5*	Ferric aerobactin receptor IutA
73	DR76_RS25915	2	IS3 family transposase
73	DR76_RS12840–	2	Intergenic region
	DR76_RS12845		
73	DR76_RS12860–	2	Intergenic region
	DR76_RS12870		
73	DR76_RS12910–	2	Intergenic region
	DR76_RS12915		
73	DR76_RS14760	2	Yersiniabactin polyketide synthase HMWP1
73	DR76_RS17040	2	Hypothetical protein
73	DR76_RS28490	4	Hypothetical protein
73	DR76_RS17640	2	Nitrate reductase molybdenum cofactor assembly chaperone
73	DR76_RS17725	2	Type VI secretion system tip protein VgrG
73	DR76_RS19605	2	Phage minor tail protein L
73	DR76_RS19790	2	DUF968 domain-containing protein
73	DR76_RS19815	2	Hypothetical protein
73	DR76_RS20430	3	Hypothetical protein
73	DR76_RS23550	3	Mechanosensitive channel MscK
73	DR76_RS24300	2	Helix-turn-helix transcriptional regulator
73	DR76_RS24705	2	Ribokinase

Loci correspond to loci in reference genomes used to annotate SNPs for each ST (see section “Materials and Methods” and “Supplementary Materials”). Loci separated by a dash (-) represent intergenic regions. *Two of the five mutations found in this gene occur at the same position.

## Discussion

The primary aims of this study were to investigate the natural history of *E. coli* infection in CF and to investigate the potential of *E. coli* patient-to-patient spread. By analyzing the genetic relationships of isolates from different patients, we were able to demonstrate that *E. coli* infection in our CF population is a dynamic process, with some patients displaying significant strain diversity developing during persistent infections over time, but found no suggestive evidence of isolates associated with infections being transmitted between patients. Rather, each patient carried their own independently evolving set of strains – likely acquired from separate reservoirs.

Most patients in our cohort were infected with a single *E. coli* pulsotype over the duration of their infections. Similarly, phylogenetic analysis of sequential isolates from individual patients revealed that most carry a single evolving strain over time. These data suggest that once a particular *E. coli* pulsotype/strain becomes established in the CF lung, it tends to persist, albeit not necessarily indefinitely, as most patients ultimately clear their infections. Recent studies of the natural history of other CF pathogens have suggested that intra-host reservoirs may be the source of repeated infections (Johansen et al., 2012); microaspiration of gastric contents may serve as a reservoir and source for *E. coli* in the CF airways.

We observed several patients in whom an initial *E. coli* pulsotype was superseded by a second, with the initial pulsotype never again recovered. As we did not type all isolates from all patients by PFGE, however, it is theoretically possible that we would have recovered the original pulsotype with denser typing, assuming two infecting strains would have the exact same morphotype on MacConkey agar. This observation is consistent with similar occurrences of strain replacement documented among other CF pathogens (Bernhardt et al., 2003; Duong et al., 2015). We further observed non-chronological phylogenetic relationships among sequentially collected isolates from individual patients. In a similar study of *S. aureus* transmission in CF, Ankrum and Hall (2017) also observed such relationships between isolates from individual patients and speculated that they may be suggestive of the simultaneous coexistence of multiple strains with indistinguishable morphologic appearances on agar media. While our dataset was not designed to investigate intra-patient diversity at a single time point, intra-patient *E. coli* diversity over time is evident based on our data. For example, 17 SNPs separated isolates from patients A013 collected a year apart. Although 17 SNPs is consistent with an intuitive definition of a “strain” (i.e. very closely related), it is too much, barring hypermutation, to represent linear evolution of a lineage over 1 year, given our substitution rate estimates. Thus, these two isolates either represent diversifying lineages (two distinct parts of the “cloud” of intra-patient diversity, suggesting coinfection with multiple “strains”), or a new lineage that replaced the previous year’s lineage (strain replacement over time).

Most patients (21/31) were infected by strains belonging to unique pulsotypes, suggesting no transmission occurred between these patients. The remaining 11 patients were infected with isolates belonging to three pulsotypes corresponding to known common ExPEC STs (Manges et al., 2019). While this is the first report to our knowledge of the isolation of STs 73 and 131 from the CF airways, the isolation of ST-1193 in CF has been previously reported (Crémet et al., 2013). ST-131 represented over half (6/11) of patients with shared STs (approximately 19% of our entire cohort) and was the single most abundant lineage in our cohort. CF isolates did not cluster together when compared to publicly available non-CF genomes corresponding to these STs, however, and likely represent a random sample from *E. coli* diversity within these STs. It remains to be determined whether the presence of these STs in CF is reflective of their overall prevalence in human populations or whether the CF airways are particularly susceptible to infection by these STs. However, ST-131 is highly prevalent in the Calgary region (Pitout et al., 2009; Peirano et al., 2010, 2012; Peirano and Pitout, 2014), and its abundance among individuals with shared pulsotypes/STs may be reflective of this. Most patients carrying isolates belonging to these STs also met the criteria for persistent infection (Figure 1), suggesting that (a) infection with these STs may be difficult to clear; (b) patients with persistent infections are more likely to carry isolates belonging to shared pulsotypes/STs; or c) persistence may be associated with carriage of specific known virulent STs.

Our analyses of individual patients’ isolates did not identify any suggestion of patient-to-patient transmission. Date estimates of common ancestors of isolates from different patients occurred too far back in time to represent transmission, often before patients were first infected with *E. coli*. Estimated substitution rates were consistent with those previously reported for *E. coli* and specifically the three shared STs (Reeves et al., 2011; Stoesser et al., 2016; Johnson et al., 2019) but were inconsistent with inter-patient SNP distances such that one patient’s isolates may have evolved directly from those of another. Further, the observation of tight phylogenetic clustering of individual patients’ isolates with long branches between patients is consistent with the independent acquisition and subsequent clonal expansion of unique *E. coli* lineages in each patient – a phenomenon recently reported in studies of infection dynamics of other CF pathogens (Caballero et al., 2015; Lee et al., 2017). We did not observe any instances where one patient’s *E. coli* diversity was completely contained within the diversity of another patient – an indicator of potential transmission noted in other transmission studies (Bryant et al., 2013).

In the only other epidemiological study of *E. coli* in CF we found, Barillova et al. determined that almost all patients carry their own sets of unique *E. coli* strains based on Enterobacterial Repetitive Intergenic Consensus (ERIC) PCR typing of 399 isolates from the airways of 45 CF patients; only 2/45 patients (∼4.4%) carried isolates belonging to the same ERIC-PCR profile (Barillova et al., 2014). This observation is in agreement with our findings that patients carry their own sets of unique strains, but we used a higher resolution approach to come to this conclusion (i.e. SNP-level analysis). Prior to the advent of WGS, the identification of patients with shared pulsotypes/STs was postulated to represent the transmission of other non-classical pathogens (Lambiase et al., 2011). According to data at this level of discrimination, we would have incorrectly suggested that approximately a third of the patients in our cohort carried *E. coli* due to transmission, when in fact independent acquisition is evident. Whether these observations apply to organisms associated with environmental reservoirs (e.g. *Stenotrophomonas maltophilia and Achromobacter* spp.) as opposed to evolved human pathogens remains to be determined. In further agreement with Barillova et al., we recovered the same strain over time from most patients with multiple infections (Barillova et al., 2014). Similarly, all shared pulsotypes/STs in our dataset belonged to the *E. coli* B2 phylogroup, although we did not determine phylogroup membership of our entire cohort.

A common finding in studies of other microorganisms in CF has been parallel pathoadaptation of the bacteria to the host lung environment (Lieberman et al., 2011; Caballero et al., 2015; Marvig et al., 2015; Pompilio et al., 2016; Silva et al., 2016; Khademi et al., 2019). The archetypal example of this is the adaptation of *P. aeruginosa* during chronic infection, which involves characteristic phenotypic and genetic changes such as loss of virulence factors and motility, production of biofilms, and mutations in global transcriptional regulators (Winstanley et al., 2016). We found 12 loci among our ST-131 isolates and 21 loci among our ST-73 isolates containing multiple (≥2) mutations, including an aerobactin receptor with 5 SNPs, two of which occurred at the same site relative to the reference. Mutations in genes related to iron acquisition are thought to play a role in the pathoadaptation of *P. aeruginosa* to the CF lung (Winstanley et al., 2016), and multiple mutations in iron uptake genes may suggest that the same may be occurring in *E. coli* in CF. However, iron uptake systems are also a common aspect of ExPEC virulence in non-CF contexts, and the accumulation of mutations in iron uptake loci may be due to the general requirement of ExPEC strains for iron in human infections (Sarowska et al., 2019). We did not observe an elevated rate of non-synonymous mutations among multi-mutated loci compared to loci with individual mutations, suggesting that locally elevated mutation rates may explain at least some of the multi-mutated loci (Caballero et al., 2015).

We recognize several limitations of our study. The primary limitation of our study – derived from the retrospective cohort analysis of a biobank – is that we only sampled one isolate per morphologically distinct *E. coli* colony per patient per time point, capturing limited intra-patient diversity. While it has traditionally been assumed that patients are infected with a single strain at a time, recent evidence from studies of other CF pathogens indicates that multi-strain infections can occur (Lee et al., 2017; O’Brien et al., 2017; Clark et al., 2018; Diaz Caballero et al., 2018; Azarian et al., 2019). For this to be true, however, these simultaneously independently infecting strains would have to have identical morphologic appearance on MacConkey agar, an unlikely scenario. Further, as we did not type every isolate from each patient by PFGE or MLST, our study is limited in its value for future global comparative studies of *E. coli* molecular epidemiology in both CF and non-CF settings. Second, although we did not observe significantly elevated substitution rates for any of the possible DNA base substitutions (Supplementary Table 1) nor non-synonymous or frameshift mutations in genes associated with hypermutation in *E. coli* (Supplementary Table 2), we did not specifically assay our isolates for hypermutator status. If any of our isolates were hypermutators, SNP differences we considered too great to represent transmission may in fact be consistent with transmission. However, we consider the latter to be unlikely based on our data. Lastly, while our mean substitution rate estimates were in line with those previously reported, our estimates had large 95% highest posterior density (HPD) intervals, likely due to the fact that divergence dates were estimated using data spanning a relatively short time period. Similarly, we lacked the data to estimate a substitution rate for the ST-1193 isolates, and so had to resort to a previously published estimate of a substitution rate for this sequence type.

## Conclusion

In conclusion, we have demonstrated the utility of a two-tiered approach using PFGE and WGS in understanding the natural history of CF airways infections with respect to *E. coli*. We observed that infection caused by *E. coli* in our CF cohort is a dynamic process, consistent with observations in similar studies of other CF pathogens (Bernhardt et al., 2003; Duong et al., 2015; Ankrum and Hall, 2017; Esposito et al., 2017; Lee et al., 2017), but there was no evidence of patient-to-patient transmission in our cohort. While most patients were infected with a single, unique *E. coli* lineage, we observed several instances of lineage replacement in patients with multiple separate infections. We further observed that patients infected with shared pulsotypes/STs tended to carry globally prevalent epidemic strains of *E. coli*, but that CF-specific isolates are a random sample from these lineages.

## Materials and Methods

### Patient Population

In this single-center multi-decade longitudinal retrospective cohort study, we analyzed *E. coli* isolates from all patients attending the Calgary Adult CF Clinic, which provides care to all patients in Southern Alberta, Canada, with at least one *E. coli*-positive sputum culture between January 1983 and December 2016. Patients were routinely followed quarterly and serial sputum samples are collected and analyzed for the presence of pathogens (Lam et al., 2015). All morphologically distinct isolates identified on MacConkey agar were assayed and subsequently confirmed as *E. coli* (in real-time) using standard methodologies and subsequently stored at −80°C in our comprehensive biobank. Infection was defined as having *E. coli* recovered from at least one sputum sample; we use the terms infection and colonization/carriage interchangeably. Patients were classified as having persistent infection if they had ≥3 *E. coli*-positive sputum cultures with carriage beyond 6 months and transient infection if they had ≥1 *E. coli*-positive sputum culture but did not meet the criteria for persistent infection. Infection episodes were defined as distinct when the last *E. coli* positive sputum culture of the first episode was separated by multiple years (>1) from the first *E. coli* positive sputum culture of the second episode, with multiple *E. coli*-negative sputum cultures collected in between and a unique pulsotype recovered from the subsequent episode. The study is approved by the Conjoint Health Research Ethics Board of the University of Calgary (REB-15-0854 and REB 15-2744).

### Pulsed-Field Gel Electrophoresis of Patient *E. coli* Isolates

From our biobank we identified initial, final and serial annual isolates from all patients with *E. coli* positive sputum. Viable *E. coli* isolates underwent pulsed-field gel electrophoresis (PFGE) using prior protocols adapted from Parkins et al. (2014) to assess for shared pulsotypes and strain persistence. 50U Xba1 (New England Biolabs) digested samples (4 h at 37°C) were run on 1% SeaKem Gold agarose with the following run conditions: 6V, 120°C, initial switch 6.76 s, final switch 35.38 s, total run time 19 h. Gels were stained with GelRed (Biotium 41003). Dendrograms were generated at 2.0% position tolerance and 1.5% optimization using the unweighted pair-group method with arithmetic mean method and the Sørensen-Dice similarity coefficient. In keeping with prior definitions (Tenover et al., 1995), strains with banding patterns ≥80% identical (i.e. ≤3 band differences) were considered related. For long-term infections, isolates were collected at first, last, and intermediate samples at 1–3 year intervals.

### Genomic DNA Extraction and Whole-Genome Sequencing

We defined shared strains as isolates belonging to pulsotypes identified from two or more patients. Bacterial isolates from frozen cultures were streaked on tryptic soy (TSY) broth agar plates and grown for single colonies overnight at 37°C. Single colonies were used to inoculate 2 ml overnight liquid cultures in TSY broth with shaking. Genomic DNA was extracted from 1 ml of overnight culture using the Promega Wizard^®^ Genomic DNA Purification Kit according to the manufacturer’s protocol. Illumina sequencing libraries were prepared using the Nextera XT DNA Library Prep Kit and sequenced on the Illumina MiSeq V3 (2 × 300 bp reads) or HiSeq V2 (2 × 250 bp reads) sequencers.

### Bioinformatic Analyses

Sequencing reads for all isolates were assessed for quality using FastQC (v. 0.11.8)^1^ and trimmed to remove adapters and low-quality reads using Trimmomatic (v. 0.38) (Bolger et al., 2014). STs for all isolates were determined from their trimmed reads using SRST2 (v. 0.2.0) (Inouye et al., 2014).

Publicly available *E. coli* genomes were downloaded from NCBI by searching for “Escherichia coli AND latest[filter] AND all[filter] NOT anomalous[filter]” (Supplementary Table 7), and *in silico* MLST performed using the MLST program^2^. Further publicly available ST-131 subgroup C1 genomes were downloaded from Matsumura et al. (2017) (Supplementary Table 7).

*De novo* assembly of all our isolate genomes and genomes from reference (Matsumura et al., 2017) was performed using SPAdes (v. 3.13.0) (Bankevich et al., 2012) using custom k-mer lengths (31, 55, 79, 103, and 127 bp), and *in silico* ST-131 clade typing for our ST-131 isolates was performed using ThermonucleotideBLAST (v. 2.04) (Gans and Wolinsky, 2008) using clade-specific primers obtained from Matsumura et al. (2017). Phylogenies for sequenced isolates supplemented with publicly available genomes were generated using Mashtree (v. 0.57) (Katz et al., 2019) for each ST.

SNP calling for all isolates for all STs was performed using Snippy (v. 4.3.6)^3^ against same-ST reference genomes [*E. coli* strain O25b:H4 for ST-131 (RefSeq Assembly Accession GCF_00285655.3), strain ATCC 25922 for ST-73 (RefSeq Assembly Accession GCF_000743255.1), and strain MCJCHV-1 for ST-1193 (RefSeq Assembly Accession GCF_003344465.1)]. Pseudo-whole genome alignments generated by Snippy were used as input to ClonalFrameML (v. 1.11) (Didelot and Wilson, 2015), along with maximum likelihood phylogenies generated using IQ-Tree (v. 1.6.10) (Nguyen et al., 2015) to identify recombinant regions, which were subsequently masked in the pseudo-whole genome alignments using the maskrc-svg tool (v. 0.5)^4^. Pairwise SNP distances were obtained from recombination-masked pseudo-whole genome alignments using snp-dists (v. 0.6.3)^5^. SNPs were annotated using snpEff (v. 4.3t) (Cingolani et al., 2012) against same-ST databases created from genbank files of the reference genomes described above.

Time-calibrated phylogenies were estimated using BEAST (v. 1.10.4) (Suchard et al., 2018). For STs 73 and 131, the HKY DNA substitution model and the best set of clock model and tree prior combinations as identified by generalized stepping-stone analysis was used (relaxed uncorrelated molecular clock with a coalescent constant size tree prior). For ST-1193, a strict clock with a previously published substitution rate of 4.03 × 10^–7^ SNPs/site/year (Johnson et al., 2019) and a coalescent constant population size tree prior was used, as we had too few isolates for accurate substitution rate estimation.

Final BEAST runs were performed using five MCMC chains of 200 million states each for a total of one billion MCMC states, with a 10% burn-in (100 million states). Log files were combined using the LogCombiner program, and maximum clade credibility trees generated using the TreeAnnotator program.

Mutation frequencies for all sequenced isolates were determined on a per-ST basis. The number of each type of base substitution was counted and divided by the total number of SNPs for each isolate, and a correction for the %GC content of the reference genome was applied as per (Payne et al., 2019). Mutations in genes associated with hypermutation in *E. coli* were annotated on a per-ST basis using snpEff (v. 4.3t) (Cingolani et al., 2012). Mutation frequencies between multi-mutated loci and those with only single mutations using chi-squared tests.

See Supplementary Materials file for full details of bioinformatic analyses.

## Data Availability Statement

The whole-genome sequencing datasets generated in this study can be found in the NCBI Short Read Archive (https://www.ncbi.nlm.nih.gov/sra/PRJNA589057). Publicly available genome assemblies used in this study can be accessed through GenBank/RefSeq using the accession numbers found in Supplementary Table 7.

## Author Contributions

BW, HR, and JG-W were responsible for accessing samples and the initial genotyping using PFGE. CI, DC, and MS were responsible for WGS and analysis. BE and RS were responsible for sample identification and clinical data collection. Statistical analyses were performed by CI, BW, and MS. MP, MS, RS, and JC envisioned the project. CI wrote the initial draft of the manuscript. All authors contributed to its revision. MP supervised the project and is the guarantor of this work.

## Conflict of Interest

The authors declare that the research was conducted in the absence of any commercial or financial relationships that could be construed as a potential conflict of interest.

## Footnotes

1 https://www.bioinformatics.babraham.ac.uk/projects/fastqc/

2 https://github.com/tseemann/mlst

3 https://github.com/tseemann/snippy

4 https://github.com/kwongj/maskrc-svg

5 https://github.com/tseemann/snp-dists
