# Supplementary material for: Epidemiology of E. coli in Cystic Fibrosis Airways Demonstrates the Capacity for Persistent Infection but Not Patient-Patient Transmission
Source: Front Microbiol. 2020 Mar 20;11:475. doi: 10.3389/fmicb.2020.00475 (PMC7100150; doi:10.3389/fmicb.2020.00475)
Supplement: Supplementary file 1 [file Data_Sheet_1.docx]

Supplementary Material

# Supplementary Methods

## Publicly Available *E. coli* Genomes

18,323 *E. coli* draft and complete genomes were identified and downloaded from NCBI using the following search criteria: “escherichia coli AND latest[filter] AND all[filter] NOT anomalous[filter]” (downloaded June 6, 2019).

A further 81 *E. coli* ST-131 subgroup C1 isolate sequencing reads were downloaded based on Short Read Archive (SRA) accession numbers found in (Matsumura et al., 2017) using the ‘fasterq-dump’ program in the SRA Toolkit (v. 2.9.6-1) with the ‘--split-files’ option set (Supplementary Table 3).

## Sequencing Read Quality Assessment and Trimming

The quality of sequencing reads for all our isolates was assessed using FastQC (v. 0.11.8) with default options (<https://www.bioinformatics.babraham.ac.uk/projects/fastqc/>). Sequencing reads for all our isolates were then trimmed using the read trimming tool Trimmomatic (Bolger et al., 2014) (v. 0.38) according to the following criteria: (i) removal of sequencing adapters (option “ILLUMINACLIP:NexteraPE-PE.fasta:2:30:10:8:true” using the adapters file NexteraPE-PE.fasta provided with Trimmomatic); (ii) removal of 1 extra base (option ‘CROP:300’ for MiSeq v3 sequenced isolates or ‘CROP:250’ for HiSeq v3 sequenced isolates); (iii) trimming of 3’ end of read if the average Phred quality score fell below 5 (option ‘SLIDINGWINDOW:4:5’); (iv) removal of reads shorter than 10 bp (option ‘MINLEN:10’).

## De Novo Assembly of Sequenced and Publicly Available *E. coli* Genomes

*De novo* assemblies for 35 sequenced isolates belonging to shared pulsotypes (Figure 1) were constructed from their trimmed sequencing reads using SPAdes (v. 3.13.0) (Bankevich et al., 2012) with default settings except for the following options: ‘--careful --cov-cutoff 10 -k 31,55,79,103,127’. The 81 ST-131 subgroup C1 publicly available genomes were assembled directly from their downloaded sequencing reads using SPAdes with the same options as above.

## Multi-locus Sequence Typing

In silico multi-locus sequence typing (MLST) for each of the 35 sequenced isolates was performed using the Short Read Sequence Typing 2 (SRST2) tool (Inouye et al., 2014) from quality-trimmed sequencing reads against the Achtman *E. coli* MLST scheme. The SRST2 “getmlst.py” python script was used to download the sequence type definitions from the *E. coli* MLST website (<https://pubmlst.org/escherichia>) (accessed February 8, 2019) (command ‘getmlst.py --species “Escherichia coli#1”’). *In silico* MLST of publicly-available *E. coli* genomes was performed on complete and draft assemblies downloaded from NCBI using the MLST program available at <https://github.com/tseemann/mlst>. Publicly available ST-131 genomes downloaded from NCBI were subsequently typed for ST-131 subgroup membership using *in silico* PCR (see below). In total, 358 ST-131 subgroup C1, 345 ST-73, and 57 ST-1193 publicly available genomes were identified (Supplementary Table 3).

## In silico PCR Clade Typing of ST-131 Isolates

Sequenced isolates and publicly available genomes downloaded from NCBI belonging to ST-131 were typed with *in silico* PCR to determine which subgroup (subclade) of ST-131 they belong to. Clade specific primer sequences were obtained from (Matsumura et al., 2017). *In silico* PCR was performed on de novo assemblies using the ‘ThermonucleotideBLAST’ program (v. 2.04) (Gans and Wolinsky, 2008). ThermonucleotideBLAST was run with the following options: ‘-i <primers file> -d <de novo assembly contigs fasta file> -e 50 -v F -m 0 -k T --primer-clamp 1 -o <output file>’.

## Publicly Available and Sequenced Isolate Phylogenies

For each of the 3 shared STs (ST-131 subgroup C1, ST-73, and ST-1193), phylogenies containing our sequenced isolates and publicly-available genomes were generated using Mashtree (v. 0.57) (Katz et al., 2019) from *de novo* assemblies (our sequenced isolates and genomes from (Matsumura et al., 2017)) and complete and draft assemblies downloaded from NCBI (all other publicly-available genomes). This included 18 sequenced and 358 publicly available genomes for ST-131, 13 sequenced and 345 publicly-available genomes for ST-73, and 4 sequenced and 57 publicly-available genomes for ST-1193. Mashtree was run for each ST with the following options: ‘--mindepth 0’, ‘--sketch-size 1000000’. Phylogenies were midpoint-rooted and visualized using the Interactive Tree of Life online tool (Letunic and Bork, 2016).

## Single-Nucleotide Polymorphism (SNP) Identification

Single-nucleotide polymorphisms for the 35 sequenced isolates were called against complete reference genomes of the same ST using the Snippy SNP calling pipeline (<https://github.com/tseemann/snippy>). Reference genomes used include *E. coli* strain O25b:H4 for ST-131 (RefSeq Assembly Accession GCF_00285655.3), ATCC 25922 for ST-73 (RefSeq Assembly Accession GCF_000743255.1), and MCJCHV-1 for ST-1193 (RefSeq Assembly Accession GCF_003344465.1). Snippy was run with default settings except for the minimum fraction of reads required to support a base call, which was set to 0.9 (‘--minfrac 0.9’ option). Core SNPs were identified using the ‘snippy-core’ program in the Snippy pipeline. SNPs were annotated using snpEff (v. 4.3t) (Cingolani et al., 2012) against same-ST databases created from genbank files of the reference genomes described above. Maximum likelihood phylogenies for each ST were created using pseudo-whole-genome alignments generated by snippy-core using IQ-Tree (Nguyen et al., 2015) with 10,000 ultrafast bootstraps (Hoang et al., 2018). The best-fitting evolutionary model for each tree (assessed by the Bayesian Information Criterion) was identified using the IQ-Tree ModelFinder (Kalyaanamoorthy et al., 2017). Bootstrap consensus trees, along with whole-genome SNP alignments from snippy-core, were used as input to ClonalFrameML (Didelot and Wilson, 2015) (v. 1.11) (run with default settings) to identify recombinant regions. SNPs found in recombinant regions were subsequently masked in pseudo-whole-genome alignments using the maskrc-svg tool (<https://github.com/kwongj/maskrc-svg>). A phylogeny containing all 35 sequenced isolates was constructed by calling SNPs for all isolates against the ST-131 reference genome and following the procedure described above. The phylogeny was visualized using the Interactive Tree of Life online tool (Letunic and Bork, 2016).

## Comparison of Pairwise SNP Distances

Recombination-masked core SNP alignments were extracted from recombination-masked pseudo-whole-genome alignments (generated above) using the ‘snp-sites’ tool (Page et al., 2016) with the ‘-c’ option set. Pairwise SNP distances were calculated from the recombination-masked core SNP alignments using the ‘snp-dists’ program (v. 0.6.3) (<https://github.com/tseemann/snp-dists>) with default settings.

## Time-Calibrated Phylogeny Estimation

Temporal signal (molecular clock) testing was performed for each ST individually from recombination-filtered core SNP phylogenies (generated by ClonalFrameML) using root-to-tip regression and date-randomization procedures as implemented in (Murray et al., 2016). Time-calibrated phylogenies were estimated for each ST individually from recombination-masked core SNP alignments for STs 73 131 and the recombination-masked pseudo-whole-genome alignment for ST-1193 using BEAST (Suchard et al., 2018) (v. 1.10.4). To account for invariant sites in the BEAST analyses of STs 73 and 131, proportions of each invariant base (A, T, C, G) from recombination-masked pseudo-whole-genome alignments were determined using custom Python scripts and input directly into the BEAUTI XML files for each BEAST run. For each ST, we used the HKY DNA substitution model with empirical base frequencies and with a site heterogeneity model accounting for invariant sites. For STs 131 and 73, we further performed model selection using generalized stepping-stone analysis in BEAST by first identifying the best-fitting clock model while keeping the tree prior constant (coalescent constant population size) from among the following: (i) strict clock, (ii) uncorrelated lognormal relaxed clock, (iii) uncorrelated relaxed exponential clock, and (iv) uncorrelated relaxed gamma clock. To select the best tree prior, we set the clock model to the best-fitting model (identified in the previous step) and identified the best tree-prior from among the following using an analogous procedure: (i) coalescent constant size; (ii) coalescent exponential population growth; (iii) coalescent lognormal population growth, (iv) coalescent expansion growth; (v) GMRF Bayesian skyride. All BEAST runs for model testing were performed in triplicate using chain lengths of 100 million states with a 10% (10 million state) burn-in. Traces were analyzed using Tracer (Rambaut et al., 2018) (v. 1.7.1). The best model/prior combinations for both STs 131 and 73 were an uncorrelated relaxed exponential clock with a coalescent constant population size tree prior. Lognormal distributions with means set to 2.46x10^-7^ (Stoesser et al., 2016) and 2.26x10^-7^ (Reeves et al., 2011) were used as clock rate priors for STs 131 and 73, respectively; a lognormal prior with mean 10 and standard deviation 100 was used for the coalescent constant size tree model prior for both STs.

For ST 1193, a strict clock with a fixed substitution rate of 4.03x10^-7^ (Johnson et al., 2019) and a coalescent constant population size tree prior (parameterized as above) was used, as our sample size was too small for accurate parameter estimation. Final BEAST runs for all STs were performed using five MCMC chains of 200 million states each for a total of one billion MCMC states with a 10% burn-in (100 million states). Log files were combined using the LogCombiner program, and maximum clade credibility trees were generated using the TreeAnnotator programs (the latter two part of the BEAST package). Maximum clade credibility trees were visualized and annotated in FigTree (v. 1.4.4) (<https://github.com/rambaut/figtree>) and the ‘ggtree’ R package (Yu et al., 2017).

## Mutation Frequency Analysis

Mutation frequencies for all sequenced isolates were determined per sequence type by counting the number of each possible base substitution observed using SNPs identified during SNP identification (section 1.7). Raw substitution counts were divided by the total number of SNPs observed for each isolate and corrected for the %GC content of the reference genomes as per (Payne et al., 2019). Mutation frequencies between multi-mutated genes and genes with only single mutations were compared using chi-square tests in R (v. 3.6.1) (R Core Team, 2019).

## Analysis of SNPs/Indels in Hypermutation-Associated Genes

SNPs/small indels for all isolates were annotated per ST using the same reference genomes as used for SNP calling (section 1.7) using snpEff (v. 4.3t) (Cingolani et al., 2012) and custom ST-specific databases created using the annotated reference genomes. A list of hypermutation associated genes was obtained from (Oliver and Mena, 2010) and mutations in these genes extracted using custom scripts in Python (v. 3.7.3).

# Supplementary Figures and Tables

## Supplementary Figures


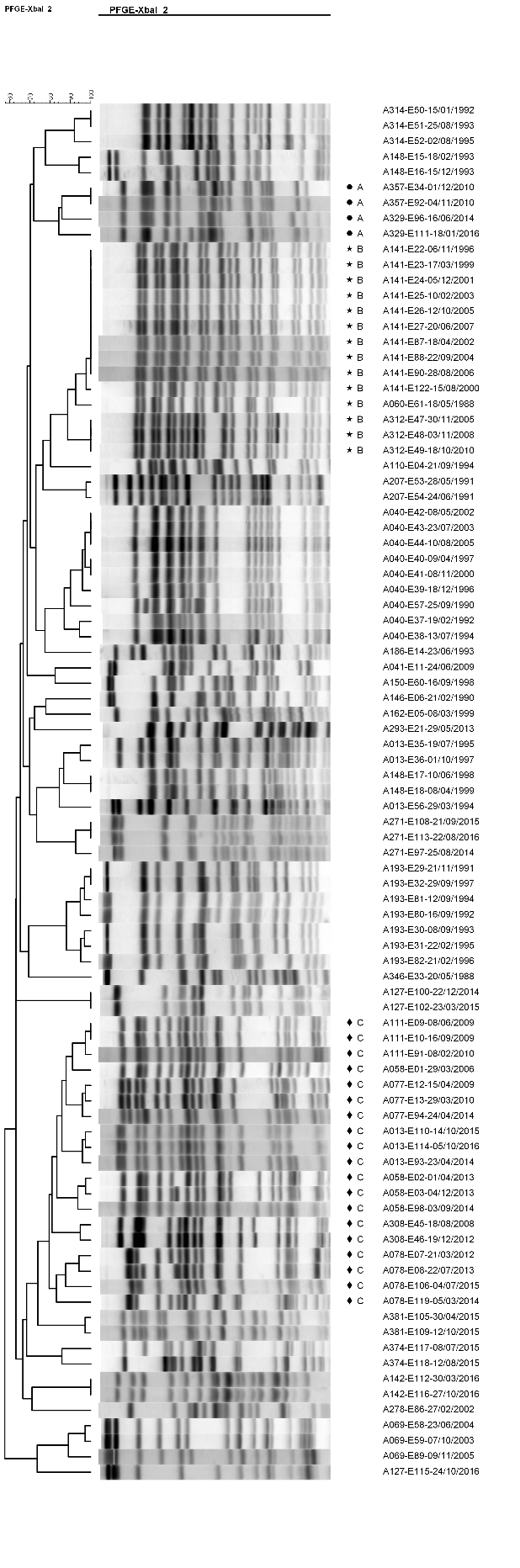


**S1 Fig. Dendrogram of 89 PFGE-typed isolates from 31 patients.** Isolates marked with a symbol and letter represent pulsotype groups shared among multiple patients. A = pulsotype cluster A (ST-1193), B = pulsotype cluster B (ST-73), C = pulsotype cluster C (ST-131). The dendrogram was generated using the UPGMA method with 2% tolerance. Isolate names are coded as “patient identifier”-“isolate number”-“culture date of isolate (dd-mm-yyyy)”. For example, A058-E01-29-03-2006 refers to isolate E01 from patient A058 cultured on March 29, 2006.


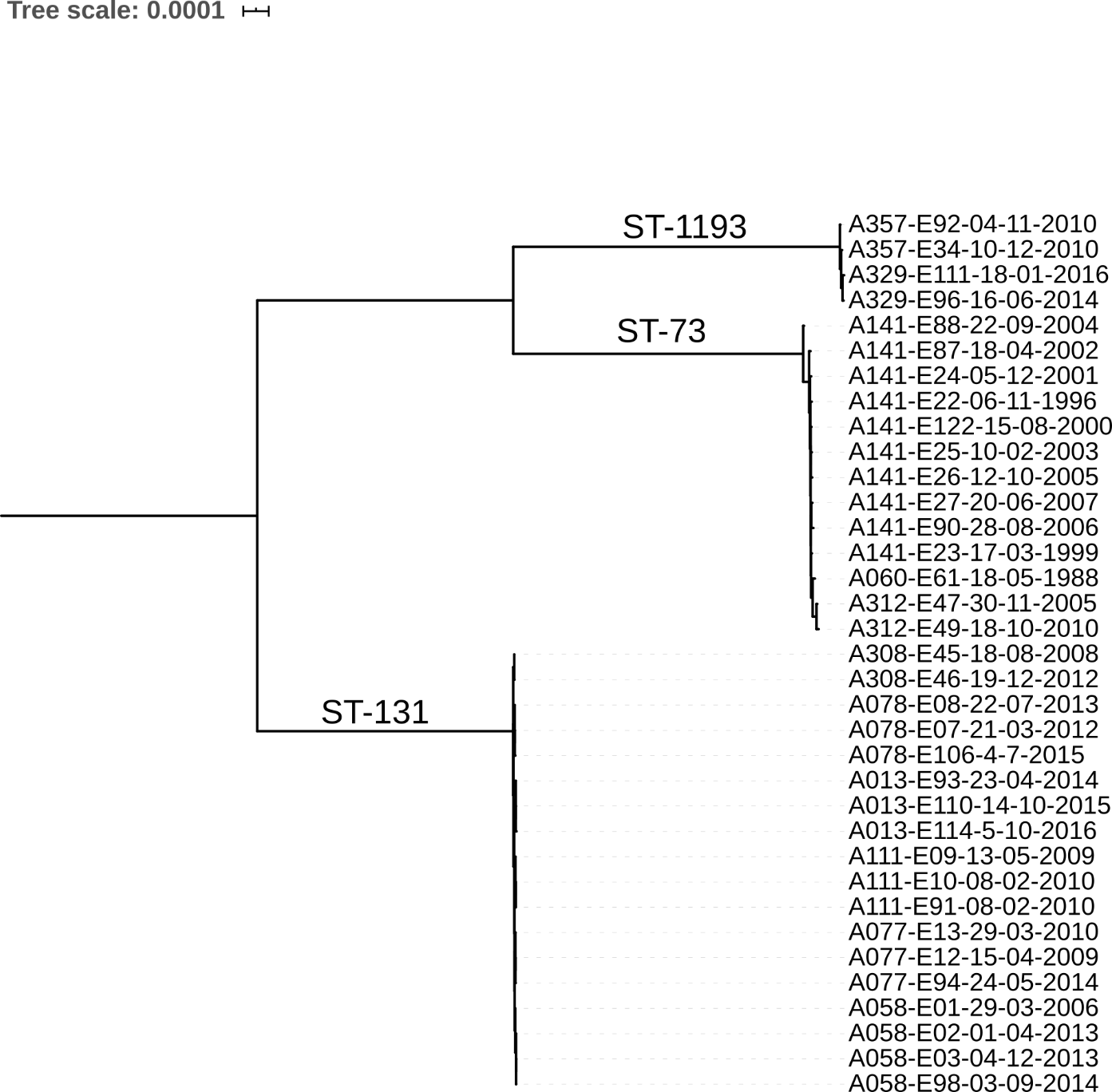


**S2 Fig. Midpoint-rooted, recombination-corrected maximum likelihood phylogeny of all 35 sequenced *E. coli* isolates.** MLST sequence types are labelled on long branches leading to each ST’s clade.


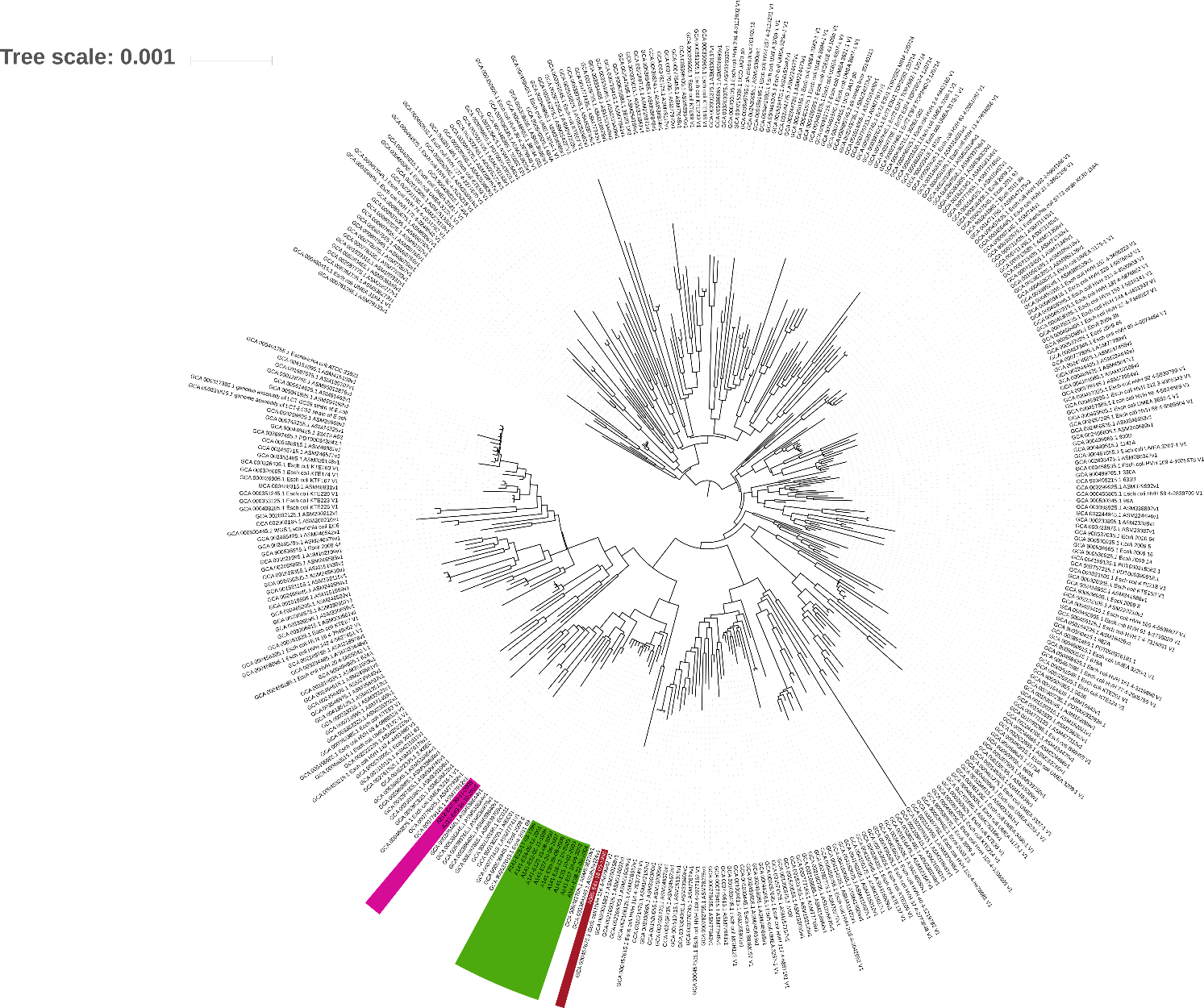


**S3 Fig. Midpoint-rooted neighbor-joining (Mashtree) phylogeny of our 13 ST-73 isolates and 345 publicly-available ST-73 genomes.** Our isolates are colored by patient: Red = patient A060, green = patient A141, pink = patient A312. Our isolate names are coded as “patient identifier”-“isolate number”-“culture date of isolate (dd-mm-yyyy)”. Publicly-available genomes are named with their RefSeq accession numbers. Label text is colored for clarity.


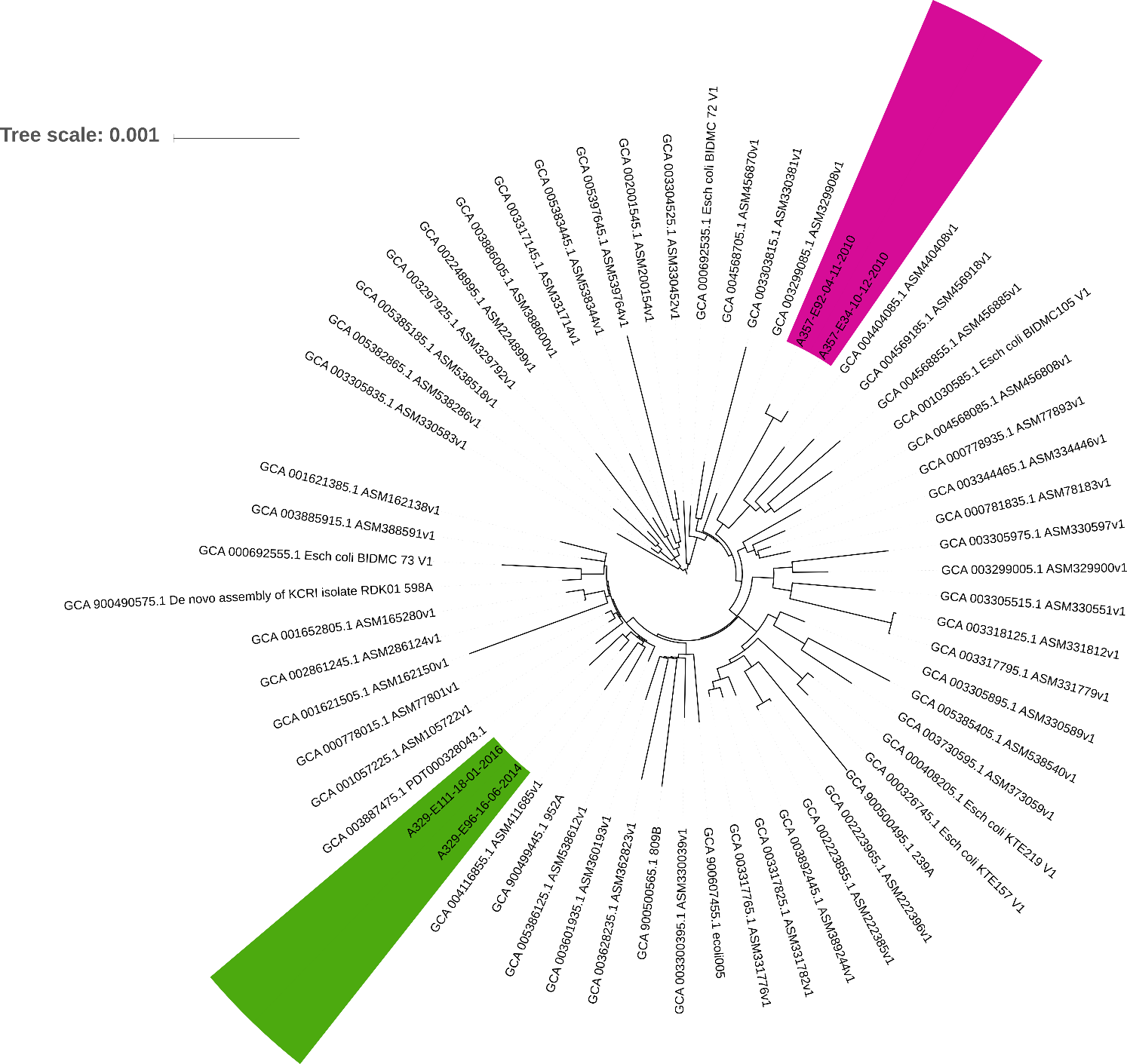


**S4 Fig.** **Midpoint-rooted neighbor-joining (Mashtree) phylogeny of our 4 ST-1193 isolates and 57 publicly-available ST-1193 genomes.** Our isolates are colored by patient: Green = patient A329, pink = patient A357. Our isolate names are coded as “patient identifier”-“isolate number”-“culture date of isolate (dd-mm-yyyy)”. Publicly-available genomes are named with their RefSeq accession numbers. Label text is colored for clarity.


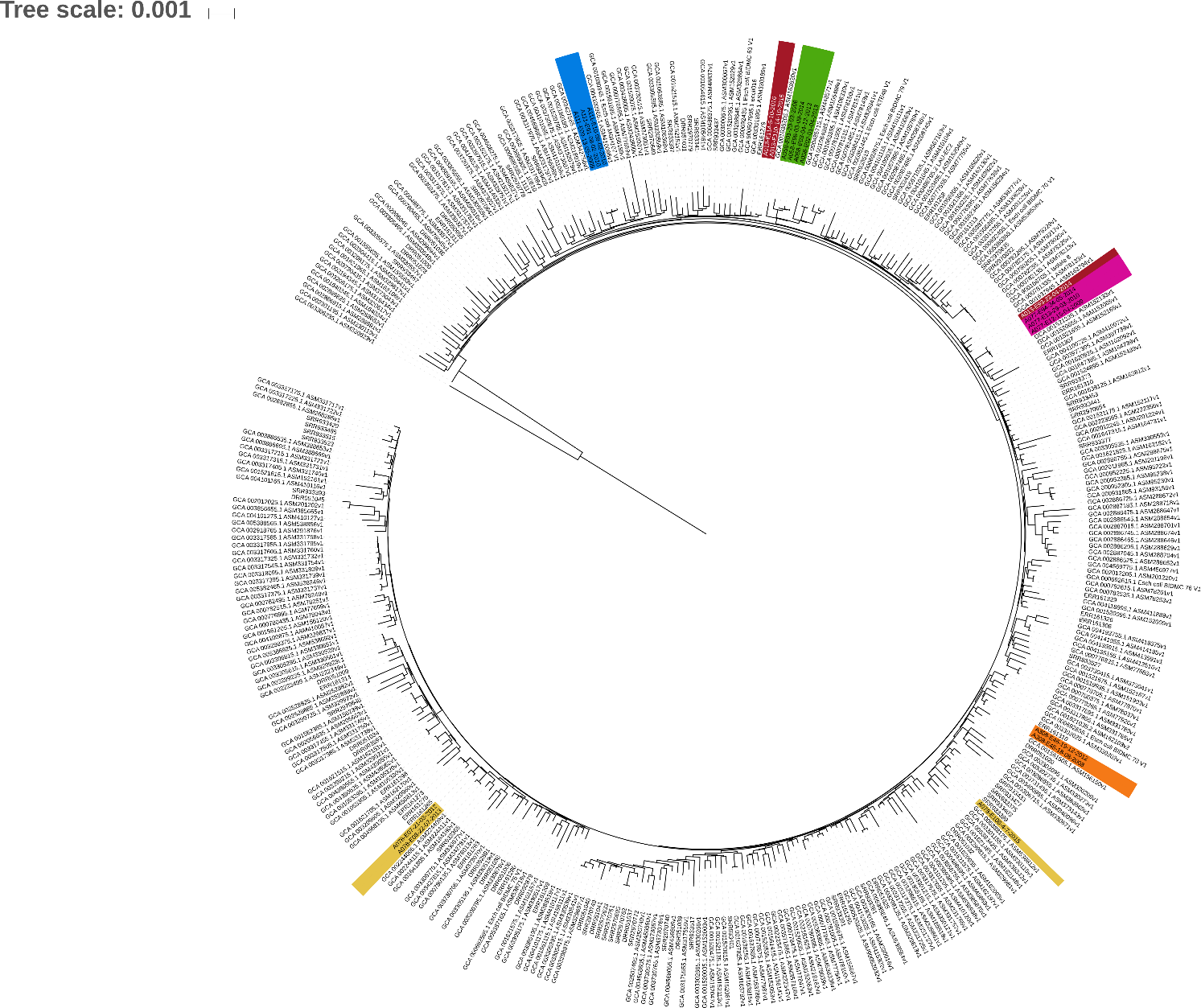


**S5 Fig.** **Midpoint-rooted neighbor-joining (Mashtree) phylogeny of our 18 ST-131 isolates and 358 publicly-available ST-131 genomes.** Our isolates are colored by patient: Blue = patient A111, red = patient A013, green = patient A058, pink = patient A077, yellow = patient A078, orange = patient A308. Our isolate names are coded as “patient identifier”-“isolate number”-“culture date of isolate (dd-mm-yyyy)”. Publicly-available genomes are named with their RefSeq or SRA accession numbers. Label text is colored for clarity.

## Supplementary Tables

**Supplementary Table 1. Mutation frequencies of sequenced *E. coli* isolates.**

| **Isolate** | **A to C** | **A to G** | **A to T** | **C to A** | **C to G** | **C to T** | **G to A** | **G to C** | **G to T** | **T to A** | **T to C** | **T to G** |
| --- | --- | --- | --- | --- | --- | --- | --- | --- | --- | --- | --- | --- |
| A111-E91-08-02-2010 | 0.0533 | 0.2133 | 0.0427 | 0.0207 | 0.0000 | 0.1345 | 0.1862 | 0.0207 | 0.0621 | 0.0320 | 0.1813 | 0.0533 |
| A013-E114-5-10-2016 | 0.0442 | 0.2036 | 0.0231 | 0.0466 | 0.0056 | 0.1546 | 0.1825 | 0.0279 | 0.0373 | 0.0327 | 0.1921 | 0.0499 |
| A111-E10-08-02-2010 | 0.0528 | 0.2111 | 0.0422 | 0.0205 | 0.0000 | 0.1331 | 0.1843 | 0.0205 | 0.0717 | 0.0317 | 0.1795 | 0.0528 |
| A077-E94-24-05-2014 | 0.0422 | 0.2042 | 0.0229 | 0.0478 | 0.0051 | 0.1331 | 0.1980 | 0.0256 | 0.0324 | 0.0317 | 0.2024 | 0.0546 |
| A058-E03-04-12-2013 | 0.0490 | 0.2023 | 0.0298 | 0.0454 | 0.0083 | 0.1383 | 0.1817 | 0.0248 | 0.0310 | 0.0383 | 0.2086 | 0.0426 |
| A308-E45-18-08-2008 | 0.0379 | 0.1921 | 0.0338 | 0.0512 | 0.0184 | 0.1587 | 0.1771 | 0.0249 | 0.0354 | 0.0365 | 0.1840 | 0.0501 |
| A308-E46-19-12-2012 | 0.0379 | 0.1965 | 0.0351 | 0.0503 | 0.0204 | 0.1551 | 0.1796 | 0.0245 | 0.0354 | 0.0337 | 0.1796 | 0.0519 |
| A077-E12-15-04-2009 | 0.0422 | 0.2024 | 0.0229 | 0.0461 | 0.0051 | 0.1366 | 0.1997 | 0.0239 | 0.0341 | 0.0317 | 0.2024 | 0.0528 |
| A058-E98-03-09-2014 | 0.0474 | 0.2019 | 0.0309 | 0.0459 | 0.0080 | 0.1418 | 0.1798 | 0.0240 | 0.0360 | 0.0350 | 0.2019 | 0.0474 |
| A078-E07-21-03-2012 | 0.0339 | 0.1878 | 0.0266 | 0.0622 | 0.0178 | 0.1590 | 0.1856 | 0.0204 | 0.0311 | 0.0366 | 0.1924 | 0.0467 |
| A013-E110-14-10-2015 | 0.0414 | 0.2009 | 0.0217 | 0.0458 | 0.0057 | 0.1566 | 0.1814 | 0.0286 | 0.0363 | 0.0354 | 0.1969 | 0.0492 |
| A078-E08-22-07-2013 | 0.0329 | 0.1935 | 0.0342 | 0.0562 | 0.0230 | 0.1595 | 0.1799 | 0.0230 | 0.0281 | 0.0329 | 0.1882 | 0.0487 |
| A111-E09-13-05-2009 | 0.0569 | 0.2048 | 0.0569 | 0.0331 | 0.0000 | 0.1214 | 0.1876 | 0.0110 | 0.0552 | 0.0341 | 0.1821 | 0.0569 |
| A077-E13-29-03-2010 | 0.0407 | 0.1881 | 0.0305 | 0.0444 | 0.0074 | 0.1356 | 0.2145 | 0.0197 | 0.0370 | 0.0280 | 0.1958 | 0.0585 |
| A013-E93-23-04-2014 | 0.0416 | 0.1981 | 0.0218 | 0.0461 | 0.0058 | 0.1536 | 0.1844 | 0.0250 | 0.0365 | 0.0337 | 0.2040 | 0.0495 |
| A058-E02-01-04-2013 | 0.0493 | 0.2036 | 0.0300 | 0.0457 | 0.0083 | 0.1392 | 0.1787 | 0.0249 | 0.0353 | 0.0343 | 0.2057 | 0.0450 |
| A078-E106-4-7-2015 | 0.0344 | 0.1839 | 0.0288 | 0.0648 | 0.0189 | 0.1675 | 0.1810 | 0.0189 | 0.0315 | 0.0371 | 0.1876 | 0.0455 |
| A058-E01-29-03-2006 | 0.0473 | 0.2094 | 0.0248 | 0.0480 | 0.0065 | 0.1332 | 0.1768 | 0.0240 | 0.0306 | 0.0360 | 0.2161 | 0.0473 |
| A141-E25-10-02-2003 | 0.0390 | 0.1704 | 0.0309 | 0.0417 | 0.0181 | 0.1899 | 0.1695 | 0.0189 | 0.0468 | 0.0305 | 0.2009 | 0.0434 |
| A141-E27-20-06-2007 | 0.0403 | 0.1701 | 0.0323 | 0.0418 | 0.0189 | 0.1906 | 0.1720 | 0.0197 | 0.0446 | 0.0306 | 0.1979 | 0.0411 |
| A141-E26-12-10-2005 | 0.0388 | 0.1701 | 0.0315 | 0.0423 | 0.0190 | 0.1898 | 0.1728 | 0.0190 | 0.0467 | 0.0295 | 0.1976 | 0.0428 |
| A060-E61-18-05-1988 | 0.0405 | 0.1748 | 0.0328 | 0.0384 | 0.0190 | 0.1897 | 0.1679 | 0.0202 | 0.0444 | 0.0312 | 0.1987 | 0.0425 |
| A141-E122-15-08-2000 | 0.0390 | 0.1683 | 0.0317 | 0.0422 | 0.0191 | 0.1910 | 0.1715 | 0.0187 | 0.0458 | 0.0301 | 0.2000 | 0.0427 |
| A141-E24-05-12-2001 | 0.0391 | 0.1668 | 0.0325 | 0.0419 | 0.0177 | 0.1943 | 0.1685 | 0.0197 | 0.0464 | 0.0309 | 0.2002 | 0.0420 |
| A312-E47-30-11-2005 | 0.0410 | 0.1751 | 0.0304 | 0.0422 | 0.0198 | 0.1794 | 0.1757 | 0.0194 | 0.0425 | 0.0318 | 0.1984 | 0.0441 |
| A312-E49-18-10-2010 | 0.0414 | 0.1699 | 0.0305 | 0.0412 | 0.0192 | 0.1835 | 0.1756 | 0.0192 | 0.0436 | 0.0327 | 0.1987 | 0.0442 |
| A141-E87-18-04-2002 | 0.0395 | 0.1690 | 0.0314 | 0.0395 | 0.0191 | 0.1928 | 0.1695 | 0.0195 | 0.0465 | 0.0293 | 0.1996 | 0.0442 |
| A141-E22-06-11-1996 | 0.0398 | 0.1695 | 0.0309 | 0.0398 | 0.0199 | 0.1897 | 0.1726 | 0.0195 | 0.0461 | 0.0301 | 0.1999 | 0.0423 |
| A141-E88-22-09-2004 | 0.0355 | 0.1710 | 0.0310 | 0.0405 | 0.0189 | 0.1973 | 0.1638 | 0.0189 | 0.0458 | 0.0319 | 0.2029 | 0.0423 |
| A141-E90-28-08-2006 | 0.0390 | 0.1700 | 0.0304 | 0.0427 | 0.0193 | 0.1911 | 0.1689 | 0.0176 | 0.0457 | 0.0308 | 0.2013 | 0.0433 |
| A141-E23-17-03-1999 | 0.0384 | 0.1699 | 0.0316 | 0.0400 | 0.0194 | 0.1896 | 0.1714 | 0.0194 | 0.0467 | 0.0303 | 0.2003 | 0.0429 |
| A357-E92-04-11-2010 | 0.0331 | 0.2977 | 0.0331 | 0.0000 | 0.0323 | 0.0484 | 0.1776 | 0.0484 | 0.0646 | 0.0331 | 0.1820 | 0.0496 |
| A357-E34-10-12-2010 | 0.0496 | 0.2976 | 0.0331 | 0.0000 | 0.0323 | 0.0484 | 0.1614 | 0.0484 | 0.0646 | 0.0331 | 0.1819 | 0.0496 |
| A329-E111-18-01-2016 | 0.0977 | 0.2605 | 0.0488 | 0.0318 | 0.0159 | 0.0954 | 0.1748 | 0.0477 | 0.0159 | 0.0488 | 0.1139 | 0.0488 |
| A329-E96-16-06-2014 | 0.0348 | 0.3133 | 0.0522 | 0.0170 | 0.0170 | 0.0850 | 0.1699 | 0.0680 | 0.0340 | 0.0348 | 0.1218 | 0.0522 |

**Supplementary Table 2. List of mutations in genes associated with hypermutation in *E. coli*.**

| **Gene** | **Isolate** | **Reference Genome** | **Position of Mutation in Reference** | **Reference Base** | **Alternate Base** | **Variant Type** |
| --- | --- | --- | --- | --- | --- | --- |
| mutS | A308-E45-18-08-2008 | HG941718.1 | 2998244 | C | T | Synonymous |
| mutS | A308-E46-19-12-2012 | HG941718.1 | 2998244 | C | T | Synonymous |
| mutS | A078-E07-21-03-2012 | HG941718.1 | 2998244 | C | T | Synonymous |
| mutS | A078-E08-22-07-2013 | HG941718.1 | 2998244 | C | T | Synonymous |
| mutS | A078-E106-4-7-2015 | HG941718.1 | 2998244 | C | T | Synonymous |
| uvrD | A141-E25-10-02-2003 | NZ_CP009072.1 | 731434 | G | A | Missense |
| mutY | A141-E25-10-02-2003 | NZ_CP009072.1 | 1784107 | A | C | Missense |
| mutY | A141-E25-10-02-2003 | NZ_CP009072.1 | 1784607 | A | G | Missense |
| uvrD | A141-E27-20-06-2007 | NZ_CP009072.1 | 731434 | G | A | Missense |
| mutY | A141-E27-20-06-2007 | NZ_CP009072.1 | 1784107 | A | C | Missense |
| mutY | A141-E27-20-06-2007 | NZ_CP009072.1 | 1784607 | A | G | Missense |
| uvrD | A141-E26-12-10-2005 | NZ_CP009072.1 | 731434 | G | A | Missense |
| mutY | A141-E26-12-10-2005 | NZ_CP009072.1 | 1784107 | A | C | Missense |
| mutY | A141-E26-12-10-2005 | NZ_CP009072.1 | 1784607 | A | G | Missense |
| uvrD | A060-E61-18-05-1988 | NZ_CP009072.1 | 731434 | G | A | Missense |
| mutY | A060-E61-18-05-1988 | NZ_CP009072.1 | 1784107 | A | C | Missense |
| mutY | A060-E61-18-05-1988 | NZ_CP009072.1 | 1784607 | A | G | Missense |
| uvrD | A141-E122-15-08-2000 | NZ_CP009072.1 | 731434 | G | A | Missense |
| mutY | A141-E122-15-08-2000 | NZ_CP009072.1 | 1784107 | A | C | Missense |
| mutY | A141-E122-15-08-2000 | NZ_CP009072.1 | 1784607 | A | G | Missense |
| uvrD | A141-E24-05-12-2001 | NZ_CP009072.1 | 731434 | G | A | Missense |
| mutY | A141-E24-05-12-2001 | NZ_CP009072.1 | 1784107 | A | C | Missense |
| mutY | A141-E24-05-12-2001 | NZ_CP009072.1 | 1784607 | A | G | Missense |
| uvrD | A312-E47-30-11-2005 | NZ_CP009072.1 | 731434 | G | A | Missense |
| mutY | A312-E47-30-11-2005 | NZ_CP009072.1 | 1784107 | A | C | Missense |
| mutY | A312-E47-30-11-2005 | NZ_CP009072.1 | 1784607 | A | G | Missense |
| uvrD | A312-E49-18-10-2010 | NZ_CP009072.1 | 731434 | G | A | Missense |
| mutY | A312-E49-18-10-2010 | NZ_CP009072.1 | 1784107 | A | C | Missense |
| mutY | A312-E49-18-10-2010 | NZ_CP009072.1 | 1784607 | A | G | Missense |
| uvrD | A141-E87-18-04-2002 | NZ_CP009072.1 | 731434 | G | A | Missense |
| mutY | A141-E87-18-04-2002 | NZ_CP009072.1 | 1784107 | A | C | Missense |
| mutY | A141-E87-18-04-2002 | NZ_CP009072.1 | 1784607 | A | G | Missense |
| uvrD | A141-E22-06-11-1996 | NZ_CP009072.1 | 731434 | G | A | Missense |
| mutY | A141-E22-06-11-1996 | NZ_CP009072.1 | 1784107 | A | C | Missense |
| mutY | A141-E22-06-11-1996 | NZ_CP009072.1 | 1784607 | A | G | Missense |
| uvrD | A141-E88-22-09-2004 | NZ_CP009072.1 | 731434 | G | A | Missense |
| mutY | A141-E88-22-09-2004 | NZ_CP009072.1 | 1784107 | A | C | Missense |
| mutY | A141-E88-22-09-2004 | NZ_CP009072.1 | 1784607 | A | G | Missense |
| uvrD | A141-E90-28-08-2006 | NZ_CP009072.1 | 731434 | G | A | Missense |
| mutY | A141-E90-28-08-2006 | NZ_CP009072.1 | 1784107 | A | C | Missense |
| mutY | A141-E90-28-08-2006 | NZ_CP009072.1 | 1784607 | A | G | Missense |
| uvrD | A141-E23-17-03-1999 | NZ_CP009072.1 | 731434 | G | A | Missense |
| mutY | A141-E23-17-03-1999 | NZ_CP009072.1 | 1784107 | A | C | Missense |
| mutY | A141-E23-17-03-1999 | NZ_CP009072.1 | 1784607 | A | G | Missense |

**Supplementary Table 3: Intra-patient (yellow) and inter-patient SNP differences for all pairwise isolate comparisons for all ST-131 subgroup C1 isolates.**

| **Isolate** | **A013-E110-14-10-2015** | **A013-E114-5-10-2016** | **A013-E93-23-04-2014** | **A058-E01-29-03-2006** | **A058-E02-01-04-2013** | **A058-E03-04-12-2013** | **A058-E98-03-09-2014** | **A077-E12-15-04-2009** | **A077-E13-29-03-2010** | **A077-E94-24-05-2014** | **A078-E07-21-03-2012** | **A078-E08-22-07-2013** | **A078-E106-4-7-2015** | **A111-E09-13-05-2009** | **A111-E10-08-02-2010** | **A111-E91-08-02-2010** | **A308-E45-18-08-2008** | **A308-E46-19-12-2012** |
| --- | --- | --- | --- | --- | --- | --- | --- | --- | --- | --- | --- | --- | --- | --- | --- | --- | --- | --- |
| A013-E110-14-10-2015 | 0 | 17 | 4 | 46 | 60 | 63 | 60 | 46 | 47 | 50 | 62 | 61 | 62 | 47 | 52 | 52 | 54 | 59 |
| A013-E114-5-10-2016 | 17 | 0 | 15 | 57 | 71 | 74 | 71 | 57 | 58 | 61 | 73 | 72 | 73 | 58 | 63 | 63 | 65 | 70 |
| A013-E93-23-04-2014 | 4 | 15 | 0 | 42 | 56 | 59 | 56 | 42 | 43 | 46 | 58 | 57 | 58 | 43 | 48 | 48 | 50 | 55 |
| A058-E01-29-03-2006 | 46 | 57 | 42 | 0 | 14 | 17 | 14 | 22 | 23 | 26 | 50 | 49 | 50 | 29 | 34 | 34 | 42 | 47 |
| A058-E02-01-04-2013 | 60 | 71 | 56 | 14 | 0 | 3 | 0 | 36 | 37 | 40 | 64 | 63 | 64 | 43 | 48 | 48 | 56 | 61 |
| A058-E03-04-12-2013 | 63 | 74 | 59 | 17 | 3 | 0 | 3 | 39 | 40 | 43 | 67 | 66 | 67 | 46 | 51 | 51 | 59 | 64 |
| A058-E98-03-09-2014 | 60 | 71 | 56 | 14 | 0 | 3 | 0 | 36 | 37 | 40 | 64 | 63 | 64 | 43 | 48 | 48 | 56 | 61 |
| A077-E12-15-04-2009 | 46 | 57 | 42 | 22 | 36 | 39 | 36 | 0 | 1 | 4 | 50 | 49 | 50 | 29 | 34 | 34 | 42 | 47 |
| A077-E13-29-03-2010 | 47 | 58 | 43 | 23 | 37 | 40 | 37 | 1 | 0 | 5 | 51 | 50 | 51 | 30 | 35 | 35 | 43 | 48 |
| A077-E94-24-05-2014 | 50 | 61 | 46 | 26 | 40 | 43 | 40 | 4 | 5 | 0 | 54 | 53 | 54 | 33 | 38 | 38 | 46 | 51 |
| A078-E07-21-03-2012 | 62 | 73 | 58 | 50 | 64 | 67 | 64 | 50 | 51 | 54 | 0 | 1 | 12 | 51 | 56 | 56 | 42 | 47 |
| A078-E08-22-07-2013 | 61 | 72 | 57 | 49 | 63 | 66 | 63 | 49 | 50 | 53 | 1 | 0 | 11 | 50 | 55 | 55 | 41 | 46 |
| A078-E106-4-7-2015 | 62 | 73 | 58 | 50 | 64 | 67 | 64 | 50 | 51 | 54 | 12 | 11 | 0 | 51 | 56 | 56 | 42 | 47 |
| A111-E09-13-05-2009 | 47 | 58 | 43 | 29 | 43 | 46 | 43 | 29 | 30 | 33 | 51 | 50 | 51 | 0 | 5 | 5 | 43 | 48 |
| A111-E10-08-02-2010 | 52 | 63 | 48 | 34 | 48 | 51 | 48 | 34 | 35 | 38 | 56 | 55 | 56 | 5 | 0 | 0 | 48 | 53 |
| A111-E91-08-02-2010 | 52 | 63 | 48 | 34 | 48 | 51 | 48 | 34 | 35 | 38 | 56 | 55 | 56 | 5 | 0 | 0 | 48 | 53 |
| A308-E45-18-08-2008 | 54 | 65 | 50 | 42 | 56 | 59 | 56 | 42 | 43 | 46 | 42 | 41 | 42 | 43 | 48 | 48 | 0 | 5 |
| A308-E46-19-12-2012 | 59 | 70 | 55 | 47 | 61 | 64 | 61 | 47 | 48 | 51 | 47 | 46 | 47 | 48 | 53 | 53 | 5 | 0 |

**Supplementary Table 4: Intra-patient (yellow) and inter-patient SNP differences for all pairwise isolate comparisons for all ST-73 isolates.**

| **Isolate** | **A060-E61-18-05-1988** | **A141-E122-15-08-2000** | **A141-E22-06-11-1996** | **A141-E23-17-03-1999** | **A141-E24-05-12-2001** | **A141-E25-10-02-2003** | **A141-E26-12-10-2005** | **A141-E27-20-06-2007** | **A141-E87-18-04-2002** | **A141-E88-22-09-2004** | **A141-E90-28-08-2006** | **A312-E47-30-11-2005** | **A312-E49-18-10-2010** |
| --- | --- | --- | --- | --- | --- | --- | --- | --- | --- | --- | --- | --- | --- |
| A060-E61-18-05-1988 | 0 | 58 | 50 | 53 | 59 | 58 | 67 | 69 | 60 | 72 | 73 | 86 | 118 |
| A141-E122-15-08-2000 | 58 | 0 | 8 | 5 | 3 | 2 | 11 | 13 | 4 | 16 | 17 | 101 | 133 |
| A141-E22-06-11-1996 | 50 | 8 | 0 | 3 | 9 | 8 | 17 | 19 | 10 | 22 | 23 | 94 | 126 |
| A141-E23-17-03-1999 | 53 | 5 | 3 | 0 | 6 | 5 | 14 | 16 | 7 | 19 | 20 | 97 | 129 |
| A141-E24-05-12-2001 | 59 | 3 | 9 | 6 | 0 | 1 | 12 | 14 | 3 | 17 | 18 | 103 | 135 |
| A141-E25-10-02-2003 | 58 | 2 | 8 | 5 | 1 | 0 | 11 | 13 | 2 | 16 | 17 | 102 | 134 |
| A141-E26-12-10-2005 | 67 | 11 | 17 | 14 | 12 | 11 | 0 | 6 | 13 | 9 | 10 | 111 | 143 |
| A141-E27-20-06-2007 | 69 | 13 | 19 | 16 | 14 | 13 | 6 | 0 | 15 | 7 | 6 | 113 | 145 |
| A141-E87-18-04-2002 | 60 | 4 | 10 | 7 | 3 | 2 | 13 | 15 | 0 | 16 | 17 | 104 | 136 |
| A141-E88-22-09-2004 | 72 | 16 | 22 | 19 | 17 | 16 | 9 | 7 | 16 | 0 | 9 | 116 | 148 |
| A141-E90-28-08-2006 | 73 | 17 | 23 | 20 | 18 | 17 | 10 | 6 | 17 | 9 | 0 | 117 | 149 |
| A312-E47-30-11-2005 | 86 | 101 | 94 | 97 | 103 | 102 | 111 | 113 | 104 | 116 | 117 | 0 | 46 |
| A312-E49-18-10-2010 | 118 | 133 | 126 | 129 | 135 | 134 | 143 | 145 | 136 | 148 | 149 | 46 | 0 |

**Supplementary Table 5: Intra-patient (yellow) and inter-patient SNP differences for all pairwise isolate comparisons for all ST-1193 isolates.**

| **Isolate** | **A329-E111-18-01-2016** | **A329-E96-16-06-2014** | **A357-E34-10-12-2010** | **A357-E92-04-11-2010** |
| --- | --- | --- | --- | --- |
| A329-E111-18-01-2016 | 0 | 7 | 39 | 40 |
| A329-E96-16-06-2014 | 7 | 0 | 38 | 39 |
| A357-E34-10-12-2010 | 39 | 38 | 0 | 1 |
| A357-E92-04-11-2010 | 40 | 39 | 1 | 0 |

**Supplementary Table 6: Divergence date estimates of nodes between patients.** Nodes are coded using patient numbers in Newick format. For example, (A111, (A058, A077)) refers to the most recent common ancestor of patient A111’s isolates and the isolates of patients A058 and A077.

| **ST** | **Node** | **Node Age (Year)** | **95% Highest Posterior Density** |
| --- | --- | --- | --- |
| 131 | (A058, A077) | 1998.5102 | (1981.6445, 2005.1494) |
| 131 | (A111, (A058, A077)) | 1993.8091 | (1969.6198, 2003,8638) |
| 131 | (A013, (A111, (A058, A077))) | 1987.9947 | (1953.2444, 2002.0409) |
| 131 | (A078, A308) | 1996.9513 | (1970.0291, 2007.0486) |
| 131 | ((A078, A308), (A013, (A111, (A058, A077)))) | 1975.2594 | (1911.7694, 2000.6814) |
| 73 | (A141, A312) | 1989.8044 | (1981.9179, 1994.9781) |
| 73 | (A060, (A141, A312)) | 1982.8005 | (1966.0317, 1988.378) |
| 1193 | (A329, A357) | 2002.7094 | (1999.4404, 2005.5809) |

**Supplementary Table 7: Accession numbers of all publicly available *E. coli* genomes used in this study.**

| **Accession** | **Sequence Type** | **Database** |
| --- | --- | --- |
| GCA_000351445.1 | 131 subgroup C1 | RefSeq |
| GCA_000485715.1 | 131 subgroup C1 | RefSeq |
| GCA_000488375.1 | 131 subgroup C1 | RefSeq |
| GCA_000488695.1 | 131 subgroup C1 | RefSeq |
| GCA_000488775.1 | 131 subgroup C1 | RefSeq |
| GCA_000498795.2 | 131 subgroup C1 | RefSeq |
| GCA_000692435.1 | 131 subgroup C1 | RefSeq |
| GCA_000692495.1 | 131 subgroup C1 | RefSeq |
| GCA_000692595.1 | 131 subgroup C1 | RefSeq |
| GCA_000692615.1 | 131 subgroup C1 | RefSeq |
| GCA_000692655.1 | 131 subgroup C1 | RefSeq |
| GCA_000692675.1 | 131 subgroup C1 | RefSeq |
| GCA_000699305.1 | 131 subgroup C1 | RefSeq |
| GCA_000699385.1 | 131 subgroup C1 | RefSeq |
| GCA_000776395.1 | 131 subgroup C1 | RefSeq |
| GCA_000776595.1 | 131 subgroup C1 | RefSeq |
| GCA_000776835.1 | 131 subgroup C1 | RefSeq |
| GCA_000776995.1 | 131 subgroup C1 | RefSeq |
| GCA_000777555.1 | 131 subgroup C1 | RefSeq |
| GCA_000777945.1 | 131 subgroup C1 | RefSeq |
| GCA_000778475.1 | 131 subgroup C1 | RefSeq |
| GCA_000778705.1 | 131 subgroup C1 | RefSeq |
| GCA_000779265.1 | 131 subgroup C1 | RefSeq |
| GCA_000779875.1 | 131 subgroup C1 | RefSeq |
| GCA_000780135.1 | 131 subgroup C1 | RefSeq |
| GCA_000780255.1 | 131 subgroup C1 | RefSeq |
| GCA_000780375.1 | 131 subgroup C1 | RefSeq |
| GCA_000780395.1 | 131 subgroup C1 | RefSeq |
| GCA_000780435.1 | 131 subgroup C1 | RefSeq |
| GCA_000780455.1 | 131 subgroup C1 | RefSeq |
| GCA_000781005.1 | 131 subgroup C1 | RefSeq |
| GCA_000781335.1 | 131 subgroup C1 | RefSeq |
| GCA_000781485.1 | 131 subgroup C1 | RefSeq |
| GCA_000781505.1 | 131 subgroup C1 | RefSeq |
| GCA_000781515.1 | 131 subgroup C1 | RefSeq |
| GCA_000782135.1 | 131 subgroup C1 | RefSeq |
| GCA_000782175.1 | 131 subgroup C1 | RefSeq |
| GCA_000782255.1 | 131 subgroup C1 | RefSeq |
| GCA_000782295.1 | 131 subgroup C1 | RefSeq |
| GCA_000782495.1 | 131 subgroup C1 | RefSeq |
| GCA_000782515.1 | 131 subgroup C1 | RefSeq |
| GCA_000782535.1 | 131 subgroup C1 | RefSeq |
| GCA_000782615.1 | 131 subgroup C1 | RefSeq |
| GCA_000931565.1 | 131 subgroup C1 | RefSeq |
| GCA_000952225.1 | 131 subgroup C1 | RefSeq |
| GCA_000952305.1 | 131 subgroup C1 | RefSeq |
| GCA_000952385.1 | 131 subgroup C1 | RefSeq |
| GCA_001030345.1 | 131 subgroup C1 | RefSeq |
| GCA_001053295.1 | 131 subgroup C1 | RefSeq |
| GCA_001053305.1 | 131 subgroup C1 | RefSeq |
| GCA_001054615.1 | 131 subgroup C1 | RefSeq |
| GCA_001056205.1 | 131 subgroup C1 | RefSeq |
| GCA_001057895.1 | 131 subgroup C1 | RefSeq |
| GCA_001063395.1 | 131 subgroup C1 | RefSeq |
| GCA_001063685.1 | 131 subgroup C1 | RefSeq |
| GCA_001064885.1 | 131 subgroup C1 | RefSeq |
| GCA_001519935.1 | 131 subgroup C1 | RefSeq |
| GCA_001520035.1 | 131 subgroup C1 | RefSeq |
| GCA_001520075.1 | 131 subgroup C1 | RefSeq |
| GCA_001520095.1 | 131 subgroup C1 | RefSeq |
| GCA_001520295.1 | 131 subgroup C1 | RefSeq |
| GCA_001520405.1 | 131 subgroup C1 | RefSeq |
| GCA_001520475.1 | 131 subgroup C1 | RefSeq |
| GCA_001520535.1 | 131 subgroup C1 | RefSeq |
| GCA_001520615.1 | 131 subgroup C1 | RefSeq |
| GCA_001520635.1 | 131 subgroup C1 | RefSeq |
| GCA_001520655.1 | 131 subgroup C1 | RefSeq |
| GCA_001520795.1 | 131 subgroup C1 | RefSeq |
| GCA_001521135.1 | 131 subgroup C1 | RefSeq |
| GCA_001521175.1 | 131 subgroup C1 | RefSeq |
| GCA_001521335.1 | 131 subgroup C1 | RefSeq |
| GCA_001521515.1 | 131 subgroup C1 | RefSeq |
| GCA_001521615.1 | 131 subgroup C1 | RefSeq |
| GCA_001521655.1 | 131 subgroup C1 | RefSeq |
| GCA_001521675.1 | 131 subgroup C1 | RefSeq |
| GCA_001524895.1 | 131 subgroup C1 | RefSeq |
| GCA_001555635.1 | 131 subgroup C1 | RefSeq |
| GCA_001561115.1 | 131 subgroup C1 | RefSeq |
| GCA_001561205.1 | 131 subgroup C1 | RefSeq |
| GCA_001561415.1 | 131 subgroup C1 | RefSeq |
| GCA_001561505.1 | 131 subgroup C1 | RefSeq |
| GCA_001561895.1 | 131 subgroup C1 | RefSeq |
| GCA_001562345.1 | 131 subgroup C1 | RefSeq |
| GCA_001562385.1 | 131 subgroup C1 | RefSeq |
| GCA_001566675.1 | 131 subgroup C1 | RefSeq |
| GCA_001620925.1 | 131 subgroup C1 | RefSeq |
| GCA_001620935.1 | 131 subgroup C1 | RefSeq |
| GCA_001621035.2 | 131 subgroup C1 | RefSeq |
| GCA_001621305.1 | 131 subgroup C1 | RefSeq |
| GCA_001621485.1 | 131 subgroup C1 | RefSeq |
| GCA_001621515.1 | 131 subgroup C1 | RefSeq |
| GCA_001621575.1 | 131 subgroup C1 | RefSeq |
| GCA_001621705.1 | 131 subgroup C1 | RefSeq |
| GCA_001621825.1 | 131 subgroup C1 | RefSeq |
| GCA_001621965.1 | 131 subgroup C1 | RefSeq |
| GCA_001621975.1 | 131 subgroup C1 | RefSeq |
| GCA_001637825.1 | 131 subgroup C1 | RefSeq |
| GCA_001637885.1 | 131 subgroup C1 | RefSeq |
| GCA_001637945.1 | 131 subgroup C1 | RefSeq |
| GCA_001638125.1 | 131 subgroup C1 | RefSeq |
| GCA_001638155.1 | 131 subgroup C1 | RefSeq |
| GCA_001638205.1 | 131 subgroup C1 | RefSeq |
| GCA_001638225.1 | 131 subgroup C1 | RefSeq |
| GCA_001640245.1 | 131 subgroup C1 | RefSeq |
| GCA_001641805.1 | 131 subgroup C1 | RefSeq |
| GCA_001647315.1 | 131 subgroup C1 | RefSeq |
| GCA_001647395.1 | 131 subgroup C1 | RefSeq |
| GCA_001855185.1 | 131 subgroup C1 | RefSeq |
| GCA_002011965.1 | 131 subgroup C1 | RefSeq |
| GCA_002012025.1 | 131 subgroup C1 | RefSeq |
| GCA_002012205.1 | 131 subgroup C1 | RefSeq |
| GCA_002012245.1 | 131 subgroup C1 | RefSeq |
| GCA_002012265.1 | 131 subgroup C1 | RefSeq |
| GCA_002056635.1 | 131 subgroup C1 | RefSeq |
| GCA_002087455.1 | 131 subgroup C1 | RefSeq |
| GCA_002087465.1 | 131 subgroup C1 | RefSeq |
| GCA_002152175.1 | 131 subgroup C1 | RefSeq |
| GCA_002180195.1 | 131 subgroup C1 | RefSeq |
| GCA_002181625.1 | 131 subgroup C1 | RefSeq |
| GCA_002223475.1 | 131 subgroup C1 | RefSeq |
| GCA_002223495.1 | 131 subgroup C1 | RefSeq |
| GCA_002223565.1 | 131 subgroup C1 | RefSeq |
| GCA_002244095.1 | 131 subgroup C1 | RefSeq |
| GCA_002244115.1 | 131 subgroup C1 | RefSeq |
| GCA_002507495.1 | 131 subgroup C1 | RefSeq |
| GCA_002528825.1 | 131 subgroup C1 | RefSeq |
| GCA_002528885.1 | 131 subgroup C1 | RefSeq |
| GCA_002682855.1 | 131 subgroup C1 | RefSeq |
| GCA_002886295.1 | 131 subgroup C1 | RefSeq |
| GCA_002886465.1 | 131 subgroup C1 | RefSeq |
| GCA_002886475.1 | 131 subgroup C1 | RefSeq |
| GCA_002886525.1 | 131 subgroup C1 | RefSeq |
| GCA_002886545.1 | 131 subgroup C1 | RefSeq |
| GCA_002886725.1 | 131 subgroup C1 | RefSeq |
| GCA_002886745.1 | 131 subgroup C1 | RefSeq |
| GCA_002886755.1 | 131 subgroup C1 | RefSeq |
| GCA_002886825.1 | 131 subgroup C1 | RefSeq |
| GCA_002886915.1 | 131 subgroup C1 | RefSeq |
| GCA_002887015.1 | 131 subgroup C1 | RefSeq |
| GCA_002887045.1 | 131 subgroup C1 | RefSeq |
| GCA_002887185.1 | 131 subgroup C1 | RefSeq |
| GCA_002918765.1 | 131 subgroup C1 | RefSeq |
| GCA_002996945.1 | 131 subgroup C1 | RefSeq |
| GCA_003000675.1 | 131 subgroup C1 | RefSeq |
| GCA_003175555.1 | 131 subgroup C1 | RefSeq |
| GCA_003194345.1 | 131 subgroup C1 | RefSeq |
| GCA_003206175.1 | 131 subgroup C1 | RefSeq |
| GCA_003297515.1 | 131 subgroup C1 | RefSeq |
| GCA_003298135.1 | 131 subgroup C1 | RefSeq |
| GCA_003298175.1 | 131 subgroup C1 | RefSeq |
| GCA_003298215.1 | 131 subgroup C1 | RefSeq |
| GCA_003298375.1 | 131 subgroup C1 | RefSeq |
| GCA_003298415.1 | 131 subgroup C1 | RefSeq |
| GCA_003298615.1 | 131 subgroup C1 | RefSeq |
| GCA_003298645.1 | 131 subgroup C1 | RefSeq |
| GCA_003298975.1 | 131 subgroup C1 | RefSeq |
| GCA_003299235.1 | 131 subgroup C1 | RefSeq |
| GCA_003299605.1 | 131 subgroup C1 | RefSeq |
| GCA_003299725.1 | 131 subgroup C1 | RefSeq |
| GCA_003299815.1 | 131 subgroup C1 | RefSeq |
| GCA_003300165.1 | 131 subgroup C1 | RefSeq |
| GCA_003301155.1 | 131 subgroup C1 | RefSeq |
| GCA_003301195.1 | 131 subgroup C1 | RefSeq |
| GCA_003301215.1 | 131 subgroup C1 | RefSeq |
| GCA_003301895.1 | 131 subgroup C1 | RefSeq |
| GCA_003302025.1 | 131 subgroup C1 | RefSeq |
| GCA_003302095.1 | 131 subgroup C1 | RefSeq |
| GCA_003302665.1 | 131 subgroup C1 | RefSeq |
| GCA_003302735.1 | 131 subgroup C1 | RefSeq |
| GCA_003303275.1 | 131 subgroup C1 | RefSeq |
| GCA_003304415.1 | 131 subgroup C1 | RefSeq |
| GCA_003305175.1 | 131 subgroup C1 | RefSeq |
| GCA_003305195.1 | 131 subgroup C1 | RefSeq |
| GCA_003305295.1 | 131 subgroup C1 | RefSeq |
| GCA_003305395.1 | 131 subgroup C1 | RefSeq |
| GCA_003305415.1 | 131 subgroup C1 | RefSeq |
| GCA_003305495.1 | 131 subgroup C1 | RefSeq |
| GCA_003305535.1 | 131 subgroup C1 | RefSeq |
| GCA_003305555.1 | 131 subgroup C1 | RefSeq |
| GCA_003305575.1 | 131 subgroup C1 | RefSeq |
| GCA_003305595.1 | 131 subgroup C1 | RefSeq |
| GCA_003305615.1 | 131 subgroup C1 | RefSeq |
| GCA_003306235.1 | 131 subgroup C1 | RefSeq |
| GCA_003306295.1 | 131 subgroup C1 | RefSeq |
| GCA_003306715.1 | 131 subgroup C1 | RefSeq |
| GCA_003306775.1 | 131 subgroup C1 | RefSeq |
| GCA_003306795.1 | 131 subgroup C1 | RefSeq |
| GCA_003306815.1 | 131 subgroup C1 | RefSeq |
| GCA_003317175.1 | 131 subgroup C1 | RefSeq |
| GCA_003317215.1 | 131 subgroup C1 | RefSeq |
| GCA_003317225.1 | 131 subgroup C1 | RefSeq |
| GCA_003317255.1 | 131 subgroup C1 | RefSeq |
| GCA_003317275.1 | 131 subgroup C1 | RefSeq |
| GCA_003317315.1 | 131 subgroup C1 | RefSeq |
| GCA_003317325.1 | 131 subgroup C1 | RefSeq |
| GCA_003317375.1 | 131 subgroup C1 | RefSeq |
| GCA_003317385.1 | 131 subgroup C1 | RefSeq |
| GCA_003317395.1 | 131 subgroup C1 | RefSeq |
| GCA_003317405.1 | 131 subgroup C1 | RefSeq |
| GCA_003317455.1 | 131 subgroup C1 | RefSeq |
| GCA_003317505.1 | 131 subgroup C1 | RefSeq |
| GCA_003317545.1 | 131 subgroup C1 | RefSeq |
| GCA_003317575.1 | 131 subgroup C1 | RefSeq |
| GCA_003317585.1 | 131 subgroup C1 | RefSeq |
| GCA_003317605.1 | 131 subgroup C1 | RefSeq |
| GCA_003317655.1 | 131 subgroup C1 | RefSeq |
| GCA_003317815.1 | 131 subgroup C1 | RefSeq |
| GCA_003317855.1 | 131 subgroup C1 | RefSeq |
| GCA_003317875.1 | 131 subgroup C1 | RefSeq |
| GCA_003317895.1 | 131 subgroup C1 | RefSeq |
| GCA_003317955.1 | 131 subgroup C1 | RefSeq |
| GCA_003317975.1 | 131 subgroup C1 | RefSeq |
| GCA_003317985.1 | 131 subgroup C1 | RefSeq |
| GCA_003318095.1 | 131 subgroup C1 | RefSeq |
| GCA_003400965.1 | 131 subgroup C1 | RefSeq |
| GCA_003427565.1 | 131 subgroup C1 | RefSeq |
| GCA_003427815.1 | 131 subgroup C1 | RefSeq |
| GCA_003428005.1 | 131 subgroup C1 | RefSeq |
| GCA_003571665.1 | 131 subgroup C1 | RefSeq |
| GCA_003730375.1 | 131 subgroup C1 | RefSeq |
| GCA_003730415.1 | 131 subgroup C1 | RefSeq |
| GCA_003730435.1 | 131 subgroup C1 | RefSeq |
| GCA_003730515.1 | 131 subgroup C1 | RefSeq |
| GCA_003730575.1 | 131 subgroup C1 | RefSeq |
| GCA_003730705.1 | 131 subgroup C1 | RefSeq |
| GCA_003730765.1 | 131 subgroup C1 | RefSeq |
| GCA_003730875.1 | 131 subgroup C1 | RefSeq |
| GCA_003731435.1 | 131 subgroup C1 | RefSeq |
| GCA_003856655.1 | 131 subgroup C1 | RefSeq |
| GCA_003858755.1 | 131 subgroup C1 | RefSeq |
| GCA_003885195.1 | 131 subgroup C1 | RefSeq |
| GCA_003886535.1 | 131 subgroup C1 | RefSeq |
| GCA_003886655.1 | 131 subgroup C1 | RefSeq |
| GCA_003886695.1 | 131 subgroup C1 | RefSeq |
| GCA_003977395.1 | 131 subgroup C1 | RefSeq |
| GCA_004100635.1 | 131 subgroup C1 | RefSeq |
| GCA_004100725.1 | 131 subgroup C1 | RefSeq |
| GCA_004100875.1 | 131 subgroup C1 | RefSeq |
| GCA_004100965.1 | 131 subgroup C1 | RefSeq |
| GCA_004101045.1 | 131 subgroup C1 | RefSeq |
| GCA_004101065.1 | 131 subgroup C1 | RefSeq |
| GCA_004101115.1 | 131 subgroup C1 | RefSeq |
| GCA_004101165.1 | 131 subgroup C1 | RefSeq |
| GCA_004101175.1 | 131 subgroup C1 | RefSeq |
| GCA_004101205.1 | 131 subgroup C1 | RefSeq |
| GCA_004101275.1 | 131 subgroup C1 | RefSeq |
| GCA_004118895.1 | 131 subgroup C1 | RefSeq |
| GCA_004135165.1 | 131 subgroup C1 | RefSeq |
| GCA_004135915.1 | 131 subgroup C1 | RefSeq |
| GCA_004141955.1 | 131 subgroup C1 | RefSeq |
| GCA_004146335.1 | 131 subgroup C1 | RefSeq |
| GCA_004153025.1 | 131 subgroup C1 | RefSeq |
| GCA_004193755.1 | 131 subgroup C1 | RefSeq |
| GCA_004380555.1 | 131 subgroup C1 | RefSeq |
| GCA_004380625.1 | 131 subgroup C1 | RefSeq |
| GCA_004568055.1 | 131 subgroup C1 | RefSeq |
| GCA_004568135.1 | 131 subgroup C1 | RefSeq |
| GCA_004568275.1 | 131 subgroup C1 | RefSeq |
| GCA_004568605.1 | 131 subgroup C1 | RefSeq |
| GCA_004569165.1 | 131 subgroup C1 | RefSeq |
| GCA_004569695.1 | 131 subgroup C1 | RefSeq |
| GCA_004569775.1 | 131 subgroup C1 | RefSeq |
| GCA_005382465.1 | 131 subgroup C1 | RefSeq |
| GCA_005383365.1 | 131 subgroup C1 | RefSeq |
| GCA_005383425.1 | 131 subgroup C1 | RefSeq |
| GCA_005385365.1 | 131 subgroup C1 | RefSeq |
| GCA_005385845.1 | 131 subgroup C1 | RefSeq |
| GCA_005386925.1 | 131 subgroup C1 | RefSeq |
| GCA_005387165.1 | 131 subgroup C1 | RefSeq |
| GCA_005387775.1 | 131 subgroup C1 | RefSeq |
| GCA_005388125.1 | 131 subgroup C1 | RefSeq |
| GCA_005388565.1 | 131 subgroup C1 | RefSeq |
| GCA_005389385.1 | 131 subgroup C1 | RefSeq |
| GCA_005671035.1 | 131 subgroup C1 | RefSeq |
| GCA_900166765.1 | 131 subgroup C1 | RefSeq |
| GCA_900520325.1 | 131 subgroup C1 | RefSeq |
| GCA_900607995.1 | 131 subgroup C1 | RefSeq |
| DRR050995 | 131 subgroup C1 | SRA |
| DRR051000 | 131 subgroup C1 | SRA |
| DRR051006 | 131 subgroup C1 | SRA |
| DRR051009 | 131 subgroup C1 | SRA |
| DRR051020 | 131 subgroup C1 | SRA |
| DRR051025 | 131 subgroup C1 | SRA |
| DRR051028 | 131 subgroup C1 | SRA |
| DRR051031 | 131 subgroup C1 | SRA |
| DRR051032 | 131 subgroup C1 | SRA |
| DRR051034 | 131 subgroup C1 | SRA |
| DRR051036 | 131 subgroup C1 | SRA |
| DRR051037 | 131 subgroup C1 | SRA |
| DRR051038 | 131 subgroup C1 | SRA |
| DRR051039 | 131 subgroup C1 | SRA |
| DRR051040 | 131 subgroup C1 | SRA |
| DRR051041 | 131 subgroup C1 | SRA |
| DRR051045 | 131 subgroup C1 | SRA |
| DRR092879 | 131 subgroup C1 | SRA |
| DRR092891 | 131 subgroup C1 | SRA |
| DRR092893 | 131 subgroup C1 | SRA |
| ERR161255 | 131 subgroup C1 | SRA |
| ERR161258 | 131 subgroup C1 | SRA |
| ERR161263 | 131 subgroup C1 | SRA |
| ERR161265 | 131 subgroup C1 | SRA |
| ERR161273 | 131 subgroup C1 | SRA |
| ERR161275 | 131 subgroup C1 | SRA |
| ERR161279 | 131 subgroup C1 | SRA |
| ERR161280 | 131 subgroup C1 | SRA |
| ERR161298 | 131 subgroup C1 | SRA |
| ERR161306 | 131 subgroup C1 | SRA |
| ERR161307 | 131 subgroup C1 | SRA |
| ERR161309 | 131 subgroup C1 | SRA |
| ERR161310 | 131 subgroup C1 | SRA |
| ERR161311 | 131 subgroup C1 | SRA |
| ERR161313 | 131 subgroup C1 | SRA |
| ERR161314 | 131 subgroup C1 | SRA |
| ERR161316 | 131 subgroup C1 | SRA |
| ERR161326 | 131 subgroup C1 | SRA |
| ERR161329 | 131 subgroup C1 | SRA |
| SRR2970632 | 131 subgroup C1 | SRA |
| SRR2970633 | 131 subgroup C1 | SRA |
| SRR2970635 | 131 subgroup C1 | SRA |
| SRR2970640 | 131 subgroup C1 | SRA |
| SRR2970647 | 131 subgroup C1 | SRA |
| SRR2970648 | 131 subgroup C1 | SRA |
| SRR2970654 | 131 subgroup C1 | SRA |
| SRR2970695 | 131 subgroup C1 | SRA |
| SRR2970740 | 131 subgroup C1 | SRA |
| SRR2970749 | 131 subgroup C1 | SRA |
| SRR2970751 | 131 subgroup C1 | SRA |
| SRR2970762 | 131 subgroup C1 | SRA |
| SRR2970772 | 131 subgroup C1 | SRA |
| SRR2970778 | 131 subgroup C1 | SRA |
| SRR2970779 | 131 subgroup C1 | SRA |
| SRR933341 | 131 subgroup C1 | SRA |
| SRR933361 | 131 subgroup C1 | SRA |
| SRR933365 | 131 subgroup C1 | SRA |
| SRR933373 | 131 subgroup C1 | SRA |
| SRR933375 | 131 subgroup C1 | SRA |
| SRR933377 | 131 subgroup C1 | SRA |
| SRR933391 | 131 subgroup C1 | SRA |
| SRR933393 | 131 subgroup C1 | SRA |
| SRR933397 | 131 subgroup C1 | SRA |
| SRR933399 | 131 subgroup C1 | SRA |
| SRR933401 | 131 subgroup C1 | SRA |
| SRR933403 | 131 subgroup C1 | SRA |
| SRR933419 | 131 subgroup C1 | SRA |
| SRR933425 | 131 subgroup C1 | SRA |
| SRR933427 | 131 subgroup C1 | SRA |
| SRR933429 | 131 subgroup C1 | SRA |
| SRR933433 | 131 subgroup C1 | SRA |
| SRR933437 | 131 subgroup C1 | SRA |
| SRR933441 | 131 subgroup C1 | SRA |
| SRR933453 | 131 subgroup C1 | SRA |
| SRR933467 | 131 subgroup C1 | SRA |
| SRR933485 | 131 subgroup C1 | SRA |
| SRR933515 | 131 subgroup C1 | SRA |
| SRR933517 | 131 subgroup C1 | SRA |
| SRR933519 | 131 subgroup C1 | SRA |
| SRR933523 | 131 subgroup C1 | SRA |
| SRR933527 | 131 subgroup C1 | SRA |
| GCA_7445.1 | 73 | RefSeq |
| GCA_148365.1 | 73 | RefSeq |
| GCA_159295.1 | 73 | RefSeq |
| GCA_164295.1 | 73 | RefSeq |
| GCA_164435.1 | 73 | RefSeq |
| GCA_164575.1 | 73 | RefSeq |
| GCA_233875.1 | 73 | RefSeq |
| GCA_233895.1 | 73 | RefSeq |
| GCA_259695.1 | 73 | RefSeq |
| GCA_317395.1 | 73 | RefSeq |
| GCA_326225.1 | 73 | RefSeq |
| GCA_326325.1 | 73 | RefSeq |
| GCA_326385.1 | 73 | RefSeq |
| GCA_326405.1 | 73 | RefSeq |
| GCA_326605.1 | 73 | RefSeq |
| GCA_326665.1 | 73 | RefSeq |
| GCA_326905.1 | 73 | RefSeq |
| GCA_331615.1 | 73 | RefSeq |
| GCA_333215.1 | 73 | RefSeq |
| GCA_350865.1 | 73 | RefSeq |
| GCA_350965.1 | 73 | RefSeq |
| GCA_351045.1 | 73 | RefSeq |
| GCA_351205.1 | 73 | RefSeq |
| GCA_351245.1 | 73 | RefSeq |
| GCA_351305.1 | 73 | RefSeq |
| GCA_351485.1 | 73 | RefSeq |
| GCA_351645.1 | 73 | RefSeq |
| GCA_351825.1 | 73 | RefSeq |
| GCA_352385.1 | 73 | RefSeq |
| GCA_353025.1 | 73 | RefSeq |
| GCA_353105.1 | 73 | RefSeq |
| GCA_353125.1 | 73 | RefSeq |
| GCA_397625.1 | 73 | RefSeq |
| GCA_397645.1 | 73 | RefSeq |
| GCA_397665.1 | 73 | RefSeq |
| GCA_397705.1 | 73 | RefSeq |
| GCA_397745.1 | 73 | RefSeq |
| GCA_401755.1 | 73 | RefSeq |
| GCA_408285.1 | 73 | RefSeq |
| GCA_456025.1 | 73 | RefSeq |
| GCA_456125.1 | 73 | RefSeq |
| GCA_456185.1 | 73 | RefSeq |
| GCA_456205.1 | 73 | RefSeq |
| GCA_456285.1 | 73 | RefSeq |
| GCA_456405.1 | 73 | RefSeq |
| GCA_456485.1 | 73 | RefSeq |
| GCA_456565.1 | 73 | RefSeq |
| GCA_456605.1 | 73 | RefSeq |
| GCA_456625.1 | 73 | RefSeq |
| GCA_456865.1 | 73 | RefSeq |
| GCA_456905.1 | 73 | RefSeq |
| GCA_456965.1 | 73 | RefSeq |
| GCA_457045.1 | 73 | RefSeq |
| GCA_457085.1 | 73 | RefSeq |
| GCA_457265.1 | 73 | RefSeq |
| GCA_457325.1 | 73 | RefSeq |
| GCA_457345.1 | 73 | RefSeq |
| GCA_457385.1 | 73 | RefSeq |
| GCA_457435.1 | 73 | RefSeq |
| GCA_457515.1 | 73 | RefSeq |
| GCA_457615.1 | 73 | RefSeq |
| GCA_457675.1 | 73 | RefSeq |
| GCA_457695.1 | 73 | RefSeq |
| GCA_457755.1 | 73 | RefSeq |
| GCA_457915.1 | 73 | RefSeq |
| GCA_458095.1 | 73 | RefSeq |
| GCA_458135.1 | 73 | RefSeq |
| GCA_458215.1 | 73 | RefSeq |
| GCA_458335.1 | 73 | RefSeq |
| GCA_458355.1 | 73 | RefSeq |
| GCA_458395.1 | 73 | RefSeq |
| GCA_458415.1 | 73 | RefSeq |
| GCA_458435.1 | 73 | RefSeq |
| GCA_458535.1 | 73 | RefSeq |
| GCA_458605.1 | 73 | RefSeq |
| GCA_459135.1 | 73 | RefSeq |
| GCA_459195.1 | 73 | RefSeq |
| GCA_459295.1 | 73 | RefSeq |
| GCA_459355.1 | 73 | RefSeq |
| GCA_459395.1 | 73 | RefSeq |
| GCA_459415.1 | 73 | RefSeq |
| GCA_459515.1 | 73 | RefSeq |
| GCA_459695.1 | 73 | RefSeq |
| GCA_459955.1 | 73 | RefSeq |
| GCA_460215.1 | 73 | RefSeq |
| GCA_460415.1 | 73 | RefSeq |
| GCA_460515.1 | 73 | RefSeq |
| GCA_460535.1 | 73 | RefSeq |
| GCA_460575.1 | 73 | RefSeq |
| GCA_460615.1 | 73 | RefSeq |
| GCA_460815.1 | 73 | RefSeq |
| GCA_460875.1 | 73 | RefSeq |
| GCA_460915.1 | 73 | RefSeq |
| GCA_460955.1 | 73 | RefSeq |
| GCA_460995.1 | 73 | RefSeq |
| GCA_461055.1 | 73 | RefSeq |
| GCA_461095.1 | 73 | RefSeq |
| GCA_461075.1 | 73 | RefSeq |
| GCA_461275.1 | 73 | RefSeq |
| GCA_461355.1 | 73 | RefSeq |
| GCA_461555.1 | 73 | RefSeq |
| GCA_461575.1 | 73 | RefSeq |
| GCA_461635.1 | 73 | RefSeq |
| GCA_461655.1 | 73 | RefSeq |
| GCA_461715.1 | 73 | RefSeq |
| GCA_461875.1 | 73 | RefSeq |
| GCA_463605.1 | 73 | RefSeq |
| GCA_488155.1 | 73 | RefSeq |
| GCA_488315.1 | 73 | RefSeq |
| GCA_488475.1 | 73 | RefSeq |
| GCA_488815.1 | 73 | RefSeq |
| GCA_494975.1 | 73 | RefSeq |
| GCA_506445.2 | 73 | RefSeq |
| GCA_507625.1 | 73 | RefSeq |
| GCA_599745.2 | 73 | RefSeq |
| GCA_599765.2 | 73 | RefSeq |
| GCA_614625.1 | 73 | RefSeq |
| GCA_711435.1 | 73 | RefSeq |
| GCA_711455.1 | 73 | RefSeq |
| GCA_713455.1 | 73 | RefSeq |
| GCA_713495.1 | 73 | RefSeq |
| GCA_713585.1 | 73 | RefSeq |
| GCA_714595.1 | 73 | RefSeq |
| GCA_714915.1 | 73 | RefSeq |
| GCA_715035.1 | 73 | RefSeq |
| GCA_743255.1 | 73 | RefSeq |
| GCA_776455.1 | 73 | RefSeq |
| GCA_776715.1 | 73 | RefSeq |
| GCA_776795.1 | 73 | RefSeq |
| GCA_777165.1 | 73 | RefSeq |
| GCA_777415.1 | 73 | RefSeq |
| GCA_777435.1 | 73 | RefSeq |
| GCA_777455.1 | 73 | RefSeq |
| GCA_777895.1 | 73 | RefSeq |
| GCA_778075.1 | 73 | RefSeq |
| GCA_778335.1 | 73 | RefSeq |
| GCA_778435.1 | 73 | RefSeq |
| GCA_778415.1 | 73 | RefSeq |
| GCA_779025.1 | 73 | RefSeq |
| GCA_779125.1 | 73 | RefSeq |
| GCA_779455.1 | 73 | RefSeq |
| GCA_779545.1 | 73 | RefSeq |
| GCA_779615.1 | 73 | RefSeq |
| GCA_779795.1 | 73 | RefSeq |
| GCA_781175.1 | 73 | RefSeq |
| GCA_781355.1 | 73 | RefSeq |
| GCA_782775.1 | 73 | RefSeq |
| GCA_782755.1 | 73 | RefSeq |
| GCA_800675.1 | 73 | RefSeq |
| GCA_807555.1 | 73 | RefSeq |
| GCA_807565.1 | 73 | RefSeq |
| GCA_807575.1 | 73 | RefSeq |
| GCA_807635.1 | 73 | RefSeq |
| GCA_807655.1 | 73 | RefSeq |
| GCA_1030285.1 | 73 | RefSeq |
| GCA_1030435.1 | 73 | RefSeq |
| GCA_1030445.1 | 73 | RefSeq |
| GCA_1056195.1 | 73 | RefSeq |
| GCA_1059015.1 | 73 | RefSeq |
| GCA_1306575.1 | 73 | RefSeq |
| GCA_1471755.2 | 73 | RefSeq |
| GCA_1518355.1 | 73 | RefSeq |
| GCA_1519115.1 | 73 | RefSeq |
| GCA_1519475.1 | 73 | RefSeq |
| GCA_1519595.1 | 73 | RefSeq |
| GCA_1520055.1 | 73 | RefSeq |
| GCA_1520215.1 | 73 | RefSeq |
| GCA_1520555.1 | 73 | RefSeq |
| GCA_1520895.1 | 73 | RefSeq |
| GCA_1521155.1 | 73 | RefSeq |
| GCA_1521355.1 | 73 | RefSeq |
| GCA_1562835.1 | 73 | RefSeq |
| GCA_1571575.1 | 73 | RefSeq |
| GCA_1616475.1 | 73 | RefSeq |
| GCA_1621345.1 | 73 | RefSeq |
| GCA_1621675.1 | 73 | RefSeq |
| GCA_1621995.1 | 73 | RefSeq |
| GCA_1693315.1 | 73 | RefSeq |
| GCA_1914005.1 | 73 | RefSeq |
| GCA_1997075.1 | 73 | RefSeq |
| GCA_2001585.1 | 73 | RefSeq |
| GCA_2002125.1 | 73 | RefSeq |
| GCA_2002165.1 | 73 | RefSeq |
| GCA_2087575.1 | 73 | RefSeq |
| GCA_2087775.1 | 73 | RefSeq |
| GCA_2109615.1 | 73 | RefSeq |
| GCA_2110115.1 | 73 | RefSeq |
| GCA_2166095.1 | 73 | RefSeq |
| GCA_2166025.1 | 73 | RefSeq |
| GCA_2166125.1 | 73 | RefSeq |
| GCA_2166685.1 | 73 | RefSeq |
| GCA_2166705.1 | 73 | RefSeq |
| GCA_2189715.1 | 73 | RefSeq |
| GCA_2189755.1 | 73 | RefSeq |
| GCA_2189765.1 | 73 | RefSeq |
| GCA_2189815.1 | 73 | RefSeq |
| GCA_2226425.1 | 73 | RefSeq |
| GCA_2231195.1 | 73 | RefSeq |
| GCA_2232105.1 | 73 | RefSeq |
| GCA_2243735.1 | 73 | RefSeq |
| GCA_2244465.1 | 73 | RefSeq |
| GCA_2244665.1 | 73 | RefSeq |
| GCA_2244735.1 | 73 | RefSeq |
| GCA_2244705.1 | 73 | RefSeq |
| GCA_2244775.1 | 73 | RefSeq |
| GCA_2244945.1 | 73 | RefSeq |
| GCA_2416865.1 | 73 | RefSeq |
| GCA_2456305.1 | 73 | RefSeq |
| GCA_2456495.1 | 73 | RefSeq |
| GCA_2456555.1 | 73 | RefSeq |
| GCA_2456615.1 | 73 | RefSeq |
| GCA_2456655.1 | 73 | RefSeq |
| GCA_2456735.1 | 73 | RefSeq |
| GCA_2465205.1 | 73 | RefSeq |
| GCA_2465425.1 | 73 | RefSeq |
| GCA_2465505.1 | 73 | RefSeq |
| GCA_2465715.1 | 73 | RefSeq |
| GCA_2465725.1 | 73 | RefSeq |
| GCA_2465795.1 | 73 | RefSeq |
| GCA_2465845.1 | 73 | RefSeq |
| GCA_2465855.1 | 73 | RefSeq |
| GCA_2466805.1 | 73 | RefSeq |
| GCA_2466835.1 | 73 | RefSeq |
| GCA_2474555.1 | 73 | RefSeq |
| GCA_2513305.1 | 73 | RefSeq |
| GCA_2519925.1 | 73 | RefSeq |
| GCA_2521835.1 | 73 | RefSeq |
| GCA_2522265.1 | 73 | RefSeq |
| GCA_2522665.1 | 73 | RefSeq |
| GCA_2522745.1 | 73 | RefSeq |
| GCA_2536335.1 | 73 | RefSeq |
| GCA_2538635.1 | 73 | RefSeq |
| GCA_2539085.1 | 73 | RefSeq |
| GCA_2542275.1 | 73 | RefSeq |
| GCA_2547695.1 | 73 | RefSeq |
| GCA_2761685.1 | 73 | RefSeq |
| GCA_2761765.1 | 73 | RefSeq |
| GCA_2803475.1 | 73 | RefSeq |
| GCA_2810035.1 | 73 | RefSeq |
| GCA_2861265.1 | 73 | RefSeq |
| GCA_2972295.1 | 73 | RefSeq |
| GCA_3146515.1 | 73 | RefSeq |
| GCA_3203375.1 | 73 | RefSeq |
| GCA_3297455.1 | 73 | RefSeq |
| GCA_3299925.1 | 73 | RefSeq |
| GCA_3300865.1 | 73 | RefSeq |
| GCA_3301085.1 | 73 | RefSeq |
| GCA_3301375.1 | 73 | RefSeq |
| GCA_3302495.1 | 73 | RefSeq |
| GCA_3304085.1 | 73 | RefSeq |
| GCA_3306575.1 | 73 | RefSeq |
| GCA_3306595.1 | 73 | RefSeq |
| GCA_3306615.1 | 73 | RefSeq |
| GCA_3333615.1 | 73 | RefSeq |
| GCA_3334095.1 | 73 | RefSeq |
| GCA_3334485.1 | 73 | RefSeq |
| GCA_3334495.1 | 73 | RefSeq |
| GCA_3334555.1 | 73 | RefSeq |
| GCA_3334615.1 | 73 | RefSeq |
| GCA_3351485.1 | 73 | RefSeq |
| GCA_3352325.1 | 73 | RefSeq |
| GCA_3388925.1 | 73 | RefSeq |
| GCA_3546975.1 | 73 | RefSeq |
| GCA_3757225.1 | 73 | RefSeq |
| GCA_3849865.1 | 73 | RefSeq |
| GCA_3885295.1 | 73 | RefSeq |
| GCA_3886045.1 | 73 | RefSeq |
| GCA_3891455.1 | 73 | RefSeq |
| GCA_3896655.1 | 73 | RefSeq |
| GCA_3897455.1 | 73 | RefSeq |
| GCA_3907235.1 | 73 | RefSeq |
| GCA_4101085.1 | 73 | RefSeq |
| GCA_4114225.1 | 73 | RefSeq |
| GCA_4135125.1 | 73 | RefSeq |
| GCA_4150065.1 | 73 | RefSeq |
| GCA_4150075.1 | 73 | RefSeq |
| GCA_4150095.1 | 73 | RefSeq |
| GCA_4150105.1 | 73 | RefSeq |
| GCA_4150175.1 | 73 | RefSeq |
| GCA_4151095.1 | 73 | RefSeq |
| GCA_4159135.1 | 73 | RefSeq |
| GCA_4522325.1 | 73 | RefSeq |
| GCA_4771135.1 | 73 | RefSeq |
| GCA_5041925.1 | 73 | RefSeq |
| GCA_5381965.1 | 73 | RefSeq |
| GCA_5382225.1 | 73 | RefSeq |
| GCA_5382705.1 | 73 | RefSeq |
| GCA_5382735.1 | 73 | RefSeq |
| GCA_5383205.1 | 73 | RefSeq |
| GCA_5383465.1 | 73 | RefSeq |
| GCA_5384245.1 | 73 | RefSeq |
| GCA_5386405.1 | 73 | RefSeq |
| GCA_5386445.1 | 73 | RefSeq |
| GCA_5386645.1 | 73 | RefSeq |
| GCA_5386845.1 | 73 | RefSeq |
| GCA_5386965.1 | 73 | RefSeq |
| GCA_5386985.1 | 73 | RefSeq |
| GCA_5387855.1 | 73 | RefSeq |
| GCA_5388245.1 | 73 | RefSeq |
| GCA_5388485.1 | 73 | RefSeq |
| GCA_5388685.1 | 73 | RefSeq |
| GCA_5389765.1 | 73 | RefSeq |
| GCA_5396405.1 | 73 | RefSeq |
| GCA_5397505.1 | 73 | RefSeq |
| GCA_5397565.1 | 73 | RefSeq |
| GCA_5397665.1 | 73 | RefSeq |
| GCA_900128765.1 | 73 | RefSeq |
| GCA_900130195.1 | 73 | RefSeq |
| GCA_900130205.1 | 73 | RefSeq |
| GCA_900244885.2 | 73 | RefSeq |
| GCA_900406575.1 | 73 | RefSeq |
| GCA_900448425.1 | 73 | RefSeq |
| GCA_900448555.1 | 73 | RefSeq |
| GCA_900448995.1 | 73 | RefSeq |
| GCA_900499215.1 | 73 | RefSeq |
| GCA_900499415.1 | 73 | RefSeq |
| GCA_900499515.1 | 73 | RefSeq |
| GCA_900499625.1 | 73 | RefSeq |
| GCA_900499765.1 | 73 | RefSeq |
| GCA_900499815.1 | 73 | RefSeq |
| GCA_900499865.1 | 73 | RefSeq |
| GCA_900499965.1 | 73 | RefSeq |
| GCA_900500065.1 | 73 | RefSeq |
| GCA_900500345.1 | 73 | RefSeq |
| GCA_900500355.1 | 73 | RefSeq |
| GCA_900500415.1 | 73 | RefSeq |
| GCA_900500515.1 | 73 | RefSeq |
| GCA_900500525.1 | 73 | RefSeq |
| GCA_900536905.1 | 73 | RefSeq |
| GCA_900536915.1 | 73 | RefSeq |
| GCA_900536925.1 | 73 | RefSeq |
| GCA_900536935.1 | 73 | RefSeq |
| GCA_900536945.1 | 73 | RefSeq |
| GCA_900536955.1 | 73 | RefSeq |
| GCA_900536965.1 | 73 | RefSeq |
| GCA_900536975.1 | 73 | RefSeq |
| GCA_900537005.1 | 73 | RefSeq |
| GCA_900536995.1 | 73 | RefSeq |
| GCA_900536985.1 | 73 | RefSeq |
| GCA_900537015.1 | 73 | RefSeq |
| GCA_900537025.1 | 73 | RefSeq |
| GCA_900537045.1 | 73 | RefSeq |
| GCA_900537035.1 | 73 | RefSeq |
| GCA_900635985.1 | 73 | RefSeq |
| GCA_900636215.1 | 73 | RefSeq |
| GCA_326745.1 | 1193 | RefSeq |
| GCA_408205.1 | 1193 | RefSeq |
| GCA_692535.1 | 1193 | RefSeq |
| GCA_692555.1 | 1193 | RefSeq |
| GCA_778015.1 | 1193 | RefSeq |
| GCA_778935.1 | 1193 | RefSeq |
| GCA_781835.1 | 1193 | RefSeq |
| GCA_1030585.1 | 1193 | RefSeq |
| GCA_1057225.1 | 1193 | RefSeq |
| GCA_1621385.1 | 1193 | RefSeq |
| GCA_1621505.1 | 1193 | RefSeq |
| GCA_1652805.1 | 1193 | RefSeq |
| GCA_2001545.1 | 1193 | RefSeq |
| GCA_2223855.1 | 1193 | RefSeq |
| GCA_2223965.1 | 1193 | RefSeq |
| GCA_2248995.1 | 1193 | RefSeq |
| GCA_2861245.1 | 1193 | RefSeq |
| GCA_3297925.1 | 1193 | RefSeq |
| GCA_3299005.1 | 1193 | RefSeq |
| GCA_3299085.1 | 1193 | RefSeq |
| GCA_3300395.1 | 1193 | RefSeq |
| GCA_3303815.1 | 1193 | RefSeq |
| GCA_3304525.1 | 1193 | RefSeq |
| GCA_3305515.1 | 1193 | RefSeq |
| GCA_3305835.1 | 1193 | RefSeq |
| GCA_3305895.1 | 1193 | RefSeq |
| GCA_3305975.1 | 1193 | RefSeq |
| GCA_3317145.1 | 1193 | RefSeq |
| GCA_3317765.1 | 1193 | RefSeq |
| GCA_3317795.1 | 1193 | RefSeq |
| GCA_3317825.1 | 1193 | RefSeq |
| GCA_3318125.1 | 1193 | RefSeq |
| GCA_3344465.1 | 1193 | RefSeq |
| GCA_3601935.1 | 1193 | RefSeq |
| GCA_3628235.1 | 1193 | RefSeq |
| GCA_3730595.1 | 1193 | RefSeq |
| GCA_3885915.1 | 1193 | RefSeq |
| GCA_3886005.1 | 1193 | RefSeq |
| GCA_3887475.1 | 1193 | RefSeq |
| GCA_3892445.1 | 1193 | RefSeq |
| GCA_4116855.1 | 1193 | RefSeq |
| GCA_4404085.1 | 1193 | RefSeq |
| GCA_4568085.1 | 1193 | RefSeq |
| GCA_4568705.1 | 1193 | RefSeq |
| GCA_4568855.1 | 1193 | RefSeq |
| GCA_4569185.1 | 1193 | RefSeq |
| GCA_5382865.1 | 1193 | RefSeq |
| GCA_5383445.1 | 1193 | RefSeq |
| GCA_5385185.1 | 1193 | RefSeq |
| GCA_5385405.1 | 1193 | RefSeq |
| GCA_5386125.1 | 1193 | RefSeq |
| GCA_5397645.1 | 1193 | RefSeq |
| GCA_900490575.1 | 1193 | RefSeq |
| GCA_900499445.1 | 1193 | RefSeq |
| GCA_900500495.1 | 1193 | RefSeq |
| GCA_900500565.1 | 1193 | RefSeq |
| GCA_900607455.1 | 1193 | RefSeq |

# References

Bankevich, A., Nurk, S., Antipov, D., Gurevich, A. A., Dvorkin, M., Kulikov, A. S., et al. (2012). SPAdes: A New Genome Assembly Algorithm and Its Applications to Single-Cell Sequencing. *J Comput Biol* 19, 455–477. doi:10.1089/cmb.2012.0021.

Bolger, A. M., Lohse, M., and Usadel, B. (2014). Trimmomatic: a flexible trimmer for Illumina sequence data. *Bioinformatics* 30, 2114–2120. doi:10.1093/bioinformatics/btu170.

Cingolani, P., Platts, A., Wang, L. L., Coon, M., Nguyen, T., Wang, L., et al. (2012). A program for annotating and predicting the effects of single nucleotide polymorphisms, SnpEff. *Fly (Austin)* 6, 80–92. doi:10.4161/fly.19695.

Didelot, X., and Wilson, D. J. (2015). ClonalFrameML: Efficient Inference of Recombination in Whole Bacterial Genomes. *PLoS Comput Biol* 11. doi:10.1371/journal.pcbi.1004041.

Gans, J. D., and Wolinsky, M. (2008). Improved assay-dependent searching of nucleic acid sequence databases. *Nucleic Acids Res* 36, e74. doi:10.1093/nar/gkn301.

Hoang, D. T., Chernomor, O., von Haeseler, A., Minh, B. Q., and Vinh, L. S. (2018). UFBoot2: Improving the Ultrafast Bootstrap Approximation. *Mol Biol Evol* 35, 518–522. doi:10.1093/molbev/msx281.

Inouye, M., Dashnow, H., Raven, L.-A., Schultz, M. B., Pope, B. J., Tomita, T., et al. (2014). SRST2: Rapid genomic surveillance for public health and hospital microbiology labs. *Genome Medicine* 6, 90. doi:10.1186/s13073-014-0090-6.

Johnson, T. J., Elnekave, E., Miller, E. A., Munoz-Aguayo, J., Figueroa, C. F., Johnston, B., et al. (2019). Phylogenomic Analysis of Extraintestinal Pathogenic Escherichia coli Sequence Type 1193, an Emerging Multidrug-Resistant Clonal Group. *Antimicrobial Agents and Chemotherapy* 63, e01913-18. doi:10.1128/AAC.01913-18.

Kalyaanamoorthy, S., Minh, B. Q., Wong, T. K., von Haeseler, A., and Jermiin, L. S. (2017). ModelFinder: Fast Model Selection for Accurate Phylogenetic Estimates. *Nat Methods* 14, 587–589. doi:10.1038/nmeth.4285.

Katz, L., Griswold, T., Morrison, S., Caravas, J., Zhang, S., Bakker, H., et al. (2019). Mashtree: a rapid comparison of whole genome sequence files. *Journal of Open Source Software* 4, 1762. doi:10.21105/joss.01762.

Letunic, I., and Bork, P. (2016). Interactive tree of life (iTOL) v3: an online tool for the display and annotation of phylogenetic and other trees. *Nucleic Acids Res* 44, W242–W245. doi:10.1093/nar/gkw290.

Matsumura, Y., Pitout, J. D. D., Peirano, G., DeVinney, R., Noguchi, T., Yamamoto, M., et al. (2017). Rapid Identification of Different Escherichia coli Sequence Type 131 Clades. *Antimicrobial Agents and Chemotherapy* 61, e00179-17. doi:10.1128/AAC.00179-17.

Murray, G. G. R., Wang, F., Harrison, E. M., Paterson, G. K., Mather, A. E., Harris, S. R., et al. (2016). The effect of genetic structure on molecular dating and tests for temporal signal. *Methods in Ecology and Evolution* 7, 80–89. doi:10.1111/2041-210X.12466.

Nguyen, L.-T., Schmidt, H. A., von Haeseler, A., and Minh, B. Q. (2015). IQ-TREE: A Fast and Effective Stochastic Algorithm for Estimating Maximum-Likelihood Phylogenies. *Mol Biol Evol* 32, 268–274. doi:10.1093/molbev/msu300.

Oliver, A., and Mena, A. (2010). Bacterial hypermutation in cystic fibrosis, not only for antibiotic resistance. *Clinical Microbiology and Infection* 16, 798–808. doi:10.1111/j.1469-0691.2010.03250.x.

Page, A. J., Taylor, B., Delaney, A. J., Soares, J., Seemann, T., Keane, J. A., et al. (2016). SNP-sites: rapid efficient extraction of SNPs from multi-FASTA alignments. *Microb Genom* 2, e000056. doi:10.1099/mgen.0.000056.

Payne, M., Octavia, S., Luu, L. D. W., Sotomayor-Castillo, C., Wang, Q., Tay, A. C. Y., et al. (2019). Enhancing genomics-based outbreak detection of endemic Salmonellaenterica serovar Typhimurium using dynamic thresholds. *Microbial Genomics*. doi:10.1099/mgen.0.000310.

R Core Team (2019). *R: A Language and Environment for Statistical Computing*. Vienna, Austria: R Foundation for Statistical Computing Available at: https://www.R-project.org/.

Rambaut, A., Drummond, A. J., Xie, D., Baele, G., and Suchard, M. A. (2018). Posterior Summarization in Bayesian Phylogenetics Using Tracer 1.7. *Syst Biol* 67, 901–904. doi:10.1093/sysbio/syy032.

Reeves, P. R., Liu, B., Zhou, Z., Li, D., Guo, D., Ren, Y., et al. (2011). Rates of Mutation and Host Transmission for an Escherichia coli Clone over 3 Years. *PLoS One* 6. doi:10.1371/journal.pone.0026907.

Stoesser, N., Sheppard, A. E., Pankhurst, L., De Maio, N., Moore, C. E., Sebra, R., et al. (2016). Evolutionary History of the Global Emergence of the Escherichia coli Epidemic Clone ST131. *mBio* 7. doi:10.1128/mBio.02162-15.

Suchard, M. A., Lemey, P., Baele, G., Ayres, D. L., Drummond, A. J., and Rambaut, A. (2018). Bayesian phylogenetic and phylodynamic data integration using BEAST 1.10. *Virus Evol* 4. doi:10.1093/ve/vey016.

Yu, G., Smith, D. K., Zhu, H., Guan, Y., and Lam, T. T.-Y. (2017). ggtree: an r package for visualization and annotation of phylogenetic trees with their covariates and other associated data. *Methods in Ecology and Evolution* 8, 28–36. doi:10.1111/2041-210X.12628.
